# Notes on *Ipomoea* (Convolvulaceae) from Ecuador

**DOI:** 10.1007/s12225-024-10186-4

**Published:** 2024-11-23

**Authors:** John R. I. Wood, Pablo Muñoz-Rodríguez, Tom Wells, David A. Espinel-Ortiz, Katya Romoleroux, Carlos Eduardo Cerón Martínez, Xavier Cornejo, Robert W. Scotland

**Affiliations:** 1 Department of Biology, University of Oxford, South Parks Road, Oxford, OX1 3RB U.K.; 2 Honorary Research Associate, Royal Botanic Gardens, Kew, Richmond, Surrey TW9 3AB U.K.; 3 Department of Biodiversity, Ecology and Evolution. Faculty of Biological Sciences, Complutense University of Madrid, José Antonio Novais 12, 28040 Madrid, Spain; 4 Laboratorio de Botánica Sistemática, Herbario QCA, Facultad de Ciencias Exactas y Naturales, Pontificia Universidad Católica del Ecuador, Av. 12 de Octubre 1076 y Vicente Ramón Roca, 170525 Quito, Ecuador; 5 Herbario Padre Luis Sodiro (QPLS), Fundación Ecuatoriana Biblioteca Aurelio Espinosa. José Nogales N69-22 y Francisco Arcos 10301, Cotocollao, Quito Ecuador; 6 Bonn Institute of Organismic Biodiversity, University of Bonn, Meckenheimer Allee 170, 53115 Bonn, Germany; 7 Herbario Alfredo Paredes (QAP), Universidad Central, Quito, Ecuador; 8 Herbario GUAY, Facultad de Ciencias Naturales, Universidad de Guayaquil, Guayaquil, Ecuador

**Keywords:** Key to species, molecular systematics, new species, species reinterpretations, taxonomy.

## Abstract

Fieldwork, examination of herbarium specimens and photographic images, supported by molecular sequencing have resulted in the re-interpretation of a number of species of *Ipomoea* L. found in Ecuador and the recognition of four new species. *Ipomoea ophiodes* Standl. & Steyerm. is shown to be a distinct species from *I. regnellii* Meisn. and their contrasting distribution is mapped. An unusual variation in *I. setosa* Ker Gawl. is discussed and illustrated. It is shown that, whereas *I. velardei* O’Donell is present in the south of Ecuador, records of *I. jujuyensis* O’Donell are probably all errors for *I. quitensis* J.R.I.Wood & Cerón, which is described as a new species endemic to Ecuador. Three other endemic species all known from single locations, *I. ceronii* J.R.I.Wood & P.Muñoz, *I. condorensis* J.R.I.Wood & Scotland and *I. papyrifera* J.R.I.Wood & Scotland are described as new. The full distribution of *I. aequatoriensis* T.Wells & P.Muñoz is mapped as a result of recent fieldwork and *I. amazonica* (D.F.Austin & Staples) J.R.I.Wood & Scotland is recorded from Ecuador for the first time. Taxonomic notes, information on habitat and distribution, maps, line drawings and photographs illustrate the species discussed. A key to all 57 species of *Ipomoea* recorded from Ecuador is provided to facilitate identification.

## Introduction

During work on the ‘Foundation Monograph of *Ipomoea* (Convolvulaceae) in the New World’ (Wood *et al.* 2020), we became aware of unusual populations of plants identified as sweet potato, *Ipomoea batatas* (L.) Lam., in coastal Ecuador (Wood *et al.* 2020: 401). The study of these populations was the basis for a grant proposal to continue our work on the sweet potato and its close relatives with a special focus on these populations in Ecuador. As a consequence, a brief exploratory visit was made to Ecuador in November 2019 and a further longer visit was made in May – June 2022 when the covid epidemic was receding. These visits brought together the team working on *Ipomoea* in Oxford University with botanists from Ecuador. This paper reports on discoveries in *Ipomoea* made in the field and the herbarium during and as a consequence of these visits with the exception of those related to the Batatas Clade (Clade A3 sensu Wood *et al.* 2020), which have been published elsewhere (Muñoz-Rodríguez *et al.* 2022). These results add to and slightly modify what was published in the monograph (Wood *et al.* 2020) but it cannot be overemphasised that the publication of a monograph is only a stage in our understanding of a genus and that additional fieldwork or consultation of previously unstudied material will inevitably result in new discoveries. This situation is similar to that of other neotropical taxa that have been monographed such as *Inga* Mill., for which the monograph (Pennington 1997) has served as a necessary context for the recognition of many new subsequent species.

## Materials and Methods

We consulted collections in the herbaria in Quito (Q, QCA, QAP, QCNE), Guayaquil (GUAY) and Loja (LOJA) (herbarium acronyms follow Thiers 2024, continuously updated, where we became aware of several collections we had not seen during the preparation of the *Ipomoea* monograph (Wood *et al.* 2020). In consequence we sought and received herbarium material on loan from herbaria with relevant collections, especially Aarhus (AAU), Gothenburg (GB), Missouri (MO) and Stockholm (S). Careful examination of this material (all specimens cited below were seen, unless otherwise indicated) with that at Oxford (OXF), Kew (K) and other cited herbaria and comparison with known species revealed four previously unrecognised species and stimulated a re-evaluation of *Ipomoea regnellii* Meisn., *I. jujuyensis* O’Donell and *I. velardei* O’Donell in Ecuador. Initial conclusions were supported by field observations and collections during our 2022 field trip to Ecuador and subsequently by relevant molecular studies. Our interpretation and use of the ITS phylogeny is extremely cautious and no taxonomic decisions are based solely on it. A complete explanation of the use of our ITS phylogeny and taxonomic decisions can be read in Muñoz-Rodríguez *et al.* (2019). Photographs from different individuals provided an important additional resource. Our species concept was set out in an earlier monograph (Wood *et al.* 2020: 14 – 16) and further elaborated in the later paper (Wells *et al.* 2021), where we argue for a heuristic species concept that recognises species as clusters of closely related individuals, responding in a similar manner to comparable sets of evolutionary and ecological forces. We, therefore, use an integrated assessment of congruence in traits indicative of genotypic, phenotypic and ecotypic cohesion to identify and delimit species.

We inferred molecular phylogenies to place the putative new species in a phylogenetic context. Building on previous work on *Ipomoea* (Muñoz-Rodríguez *et al.* 2019; Jara *et al.* 2020)*,* we used the nuclear Internal Transcribed Spacer DNA barcode (*nrITS*) to place the new samples in a phylogenetic context. We sequenced four Ecuadorian samples: *J. R. I. Wood* 29602, 29603, 29610 and 29619 but were unsuccessful with older material assigned to *I. condorensis* and *I. papyrifera*, which are described below. We extracted DNA using the Qiagen DNEasy extraction kit and used primers AB101 and AB102 (Douzery *et al.* 1999) for nrITS amplification with a reagent volume of 15 μl (7.3 μl H_2_O, 3 μl buffer, 0.7 μl MgCl_2_, 0.3 μl of each primer diluted to a concentration of 10 ×, 0.5 μl dNTPs, 1 μl BSA, 0.4 μl Taq polymerase, 1.5 μl sample DNA) and standard PCR conditions (5’ at 80°C; 30 cycles of 1’ at 95°C, 1’ at 50°C, 4’ at 65°C and a final stage of 4’ at 65°C). We cleaned the PCR reaction using the GeneJET PCR purification kit. Sanger sequencing was conducted at Source Bioscience with the same primers used in the PCR. The new sequences are available via GenBank, numbers OR148057 – OR148064.

**Fig. 1.**
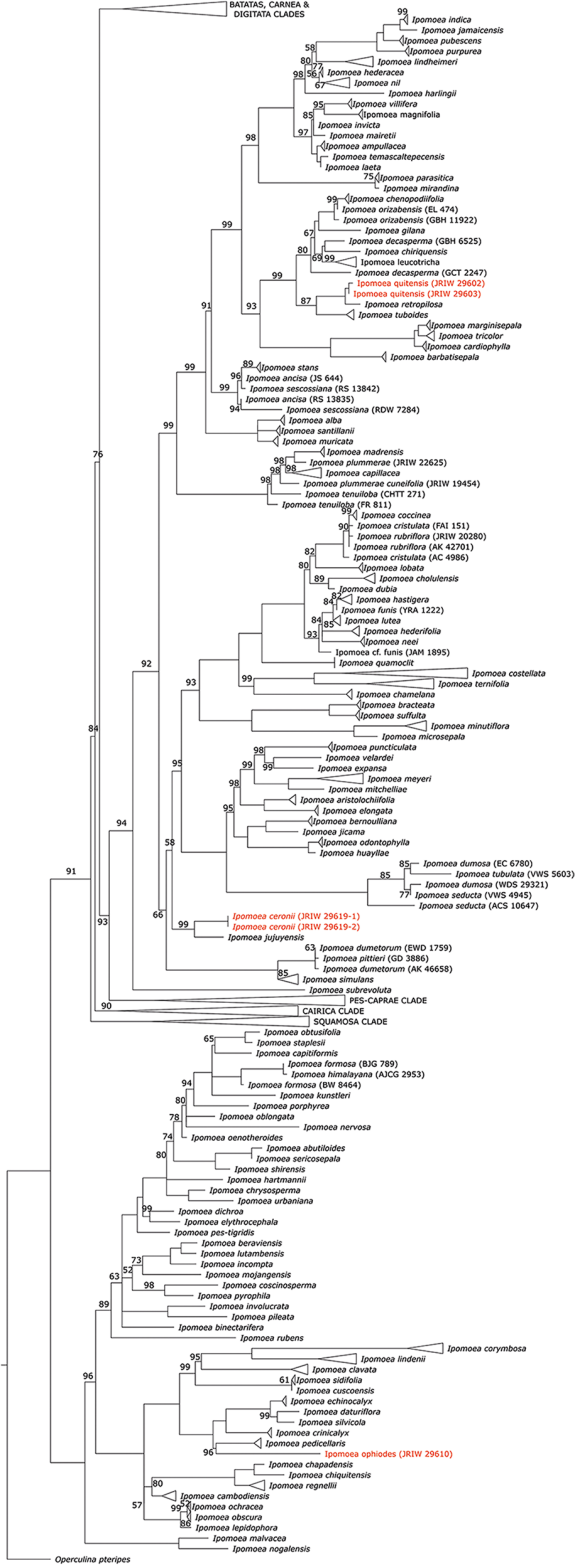
Species-level *nrITS* phylogeny of *Ipomoea,* showing the position of *I. ophiodes* and the new species *I. quitensis* and *I. ceronii*, in red. Maximum likelihood phylogeny inferred using IQ-TREE (SYM+I+G4 model). Numbers on the branches indicate support values (1000 ultrafast bootstrap replicates); branches without numbers indicate 100% support. Triangles indicate species in this phylogeny represented by more than one specimen and forming a monophyletic group, simplified here for clarity; it must be noted that non-monophyly in this phylogeny may be due to lack of resolution in this *nrITS* phylogeny, rather than actual evolutionary signal (see Muñoz-Rodríguez *et al.* 2019).

In addition to the newly sequenced samples, we used 260 sequences previously generated representing the diversity within *Ipomoea* to infer the phylogeny (Fig. 1), and an *Operculina pteripes* (G.Don) O'Donell sample as outgroup. All molecular data used in this study was uploaded to a public repository, available from: https://zenodo.org/records/10453911.

We first used default settings in NCBI Blast to confirm the sequences were good and to identify the species most closely related to each of our samples. We then inferred a phylogeny including specimens representing the phylogenetic diversity existing in *Ipomoea* worldwide. We aligned the sequences using MAFFT v.7.310 (Katoh & Standley 2013, 2016) and removed all columns in the alignment with 90% or more gaps, using Geneious v.9.1.8 (https://www.geneious.com). We then inferred a Maximum Likelihood phylogeny using IQ-TREE 2.2.2.3 (Nguyen *et al.* 2015), with automatic model selection using ModelFinder (Kalyaanamoorthy *et al.* 2017) and 1000 ultrafast bootstrap replicates (seed 44044, one thread). The SYM+I+G4 model was selected based on the Bayesian Information Criterion. In the resulting phylogeny, we collapsed all nodes with less than 60% bootstrap support into polytomies (Fig. 1).

## Results

The results of our field and herbarium studies are arranged alphabetically below under species names, with some modifications so discussion can lead logically from one species to another. Four new species are described, two species complexes are re-interpreted, one species is reported from Ecuador for the first time, another shown to be unexpectedly widespread in western parts of the country and an unusual variation reported for a third.

## Taxonomic Treatment

**Ipomoea aequatoriensis** *T.Wells & P.Muñoz*

**Map 1.**
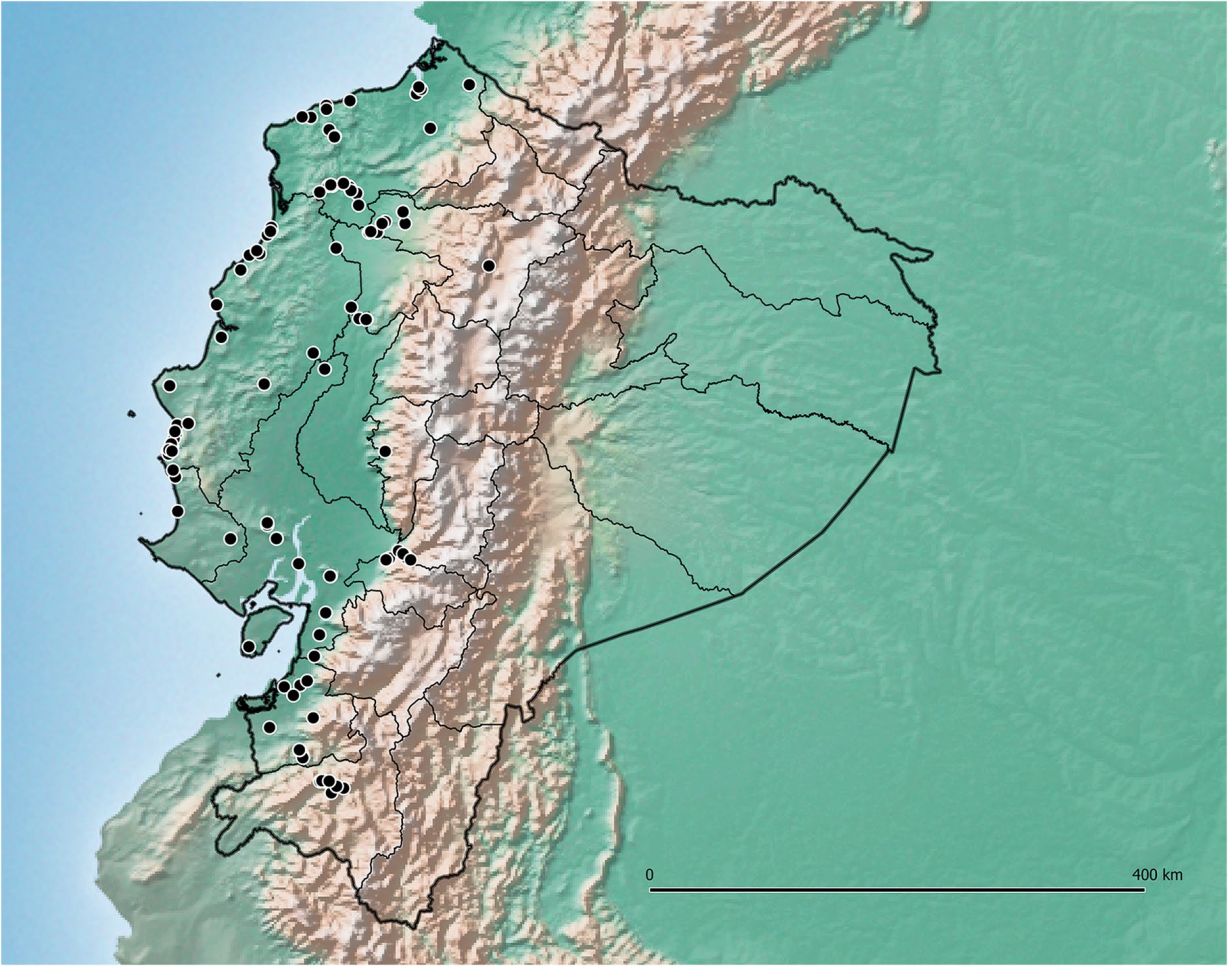
Distribution of *Ipomoea aequatoriensis.*

**Fig. 2.**
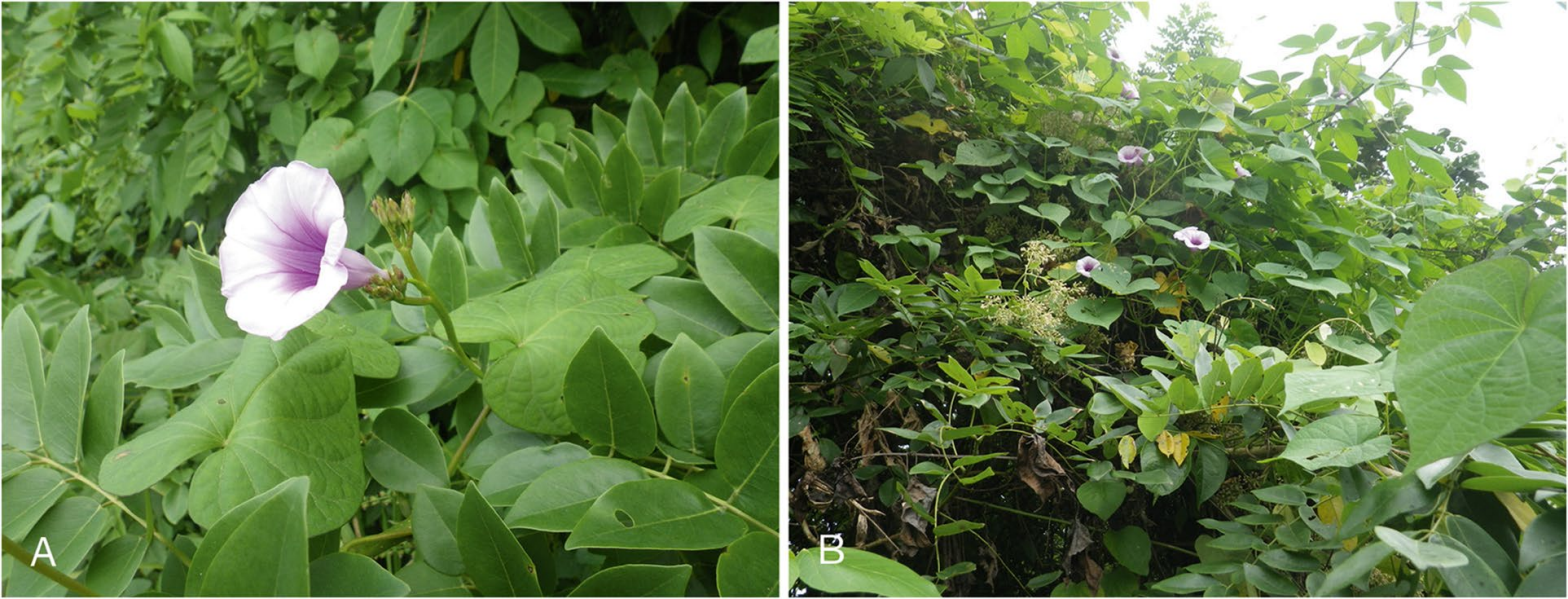
*Ipomoea aequatoriensis*. **A** flower;** B** habit. photos: john wood.

This recently described species (Muñoz-Rodríguez *et al.* 2022) proved to be unexpectedly abundant in western Ecuador up to slightly above 2000 m near Loja, although most collections were made in the lowlands (Map 1). Most, perhaps all records cited by Austin (1982) under *Ipomoea batatas* (L.) Lam. from the provinces of Esmeraldas, Manabí, Guayas, Los Ríos, El Oro and Pichincha should be referred to this species. *Ipomoea aequatoriensis* is most likely the direct descendant of the progenitor of *I. batatas* (Muñoz-Rodríguez *et al.* 2022) and the two species are not always easily distinguished, as both are variable morphologically. *Ipomoea batatas* is often found as a trailing plant in or near settlements or cultivation, whereas *I. aequatoriensis* is a more slender plant, apparently lacking large storage roots and usually scrambling or twining over scrub (Fig. 2A, B) in disturbed habitats both by roadsides and in more natural habitats away from human settlement.

**Ipomoea amazonica** (*D.F.Austin & Staples*) *J.R.I.Wood & Scotland*

The following was found in material received too late for inclusion in the monograph and represents the first record of this species in Ecuador, filling in a gap in its range around the Amazon basin.

**specimens examined. ecuador.** **Orellana:** Tiputini Biodiversity Station, Rio Tiputani, c. 20 km by air E of confluence with Rio Tivacuno, c. 8 – 10 km downstream of station along Río Tiputini, liana in floodplain forest, 200 – 300 m, 00°39'14"S 76°08'13"W, 11 Nov. 1998, *R. J. Burnham et al. *1825 (MICH, OXF).

**Ipomoea batatoides** *Choisy*

*Ipomoea batatoides* usually has pink corollas but occasional, white-flowered forms are found. The two following specimens come from protected areas in the biodiverse-rich rainforests of Amazonian Ecuador.

**specimens examined. ecuador.** **Orellana:** P. N. Yasuni, km 48 of Maxus Petroleum road, 200 – 300 m, 3 Nov. 1998, *R. J. Burnham* 1793 (MICH, OXF); Tiputini Biodiversity Station, Río Tiputini, 25 km E of confluence with Río Tivacuno, 00°39'S 76°08'W, 200 – 300 m, 29 Sept. 1998, *R. J. Burnham* 1735 (MICH, OXF).

*Burnham* 1735 is of particular interest as the leaves are pubescent, an exceptional character in *Ipomoea batatoides*. Further collections are needed to evaluate this population. White-flowered forms of *I. batatoides* occur occasionally elsewhere in the neotropics (Wood *et al.* 2020: 298). White-flowered forms are not so easily distinguished from related species found in Central America and Mexico, such as *I. pseudoracemosa* McPherson, a poorly understood Mexican species, nearly always leafless at anthesis.

**Ipomoea ceronii** *J.R.I.Wood & P.Muñoz* **sp. nov.** Type: Ecuador, Chimborazo, Huigra, 2°17'08.2"S 78°59'59.4"W, 2930 m, 2 June 2022, *J. R. I. Wood, P. Muñoz, T. Wells & D. Espinel* 29619 (holotype QCA; isotypes OXF, QAP).

http://www.ipni.org//urn:lsid:ipni.org:names:77348685-1

**Fig. 3.**
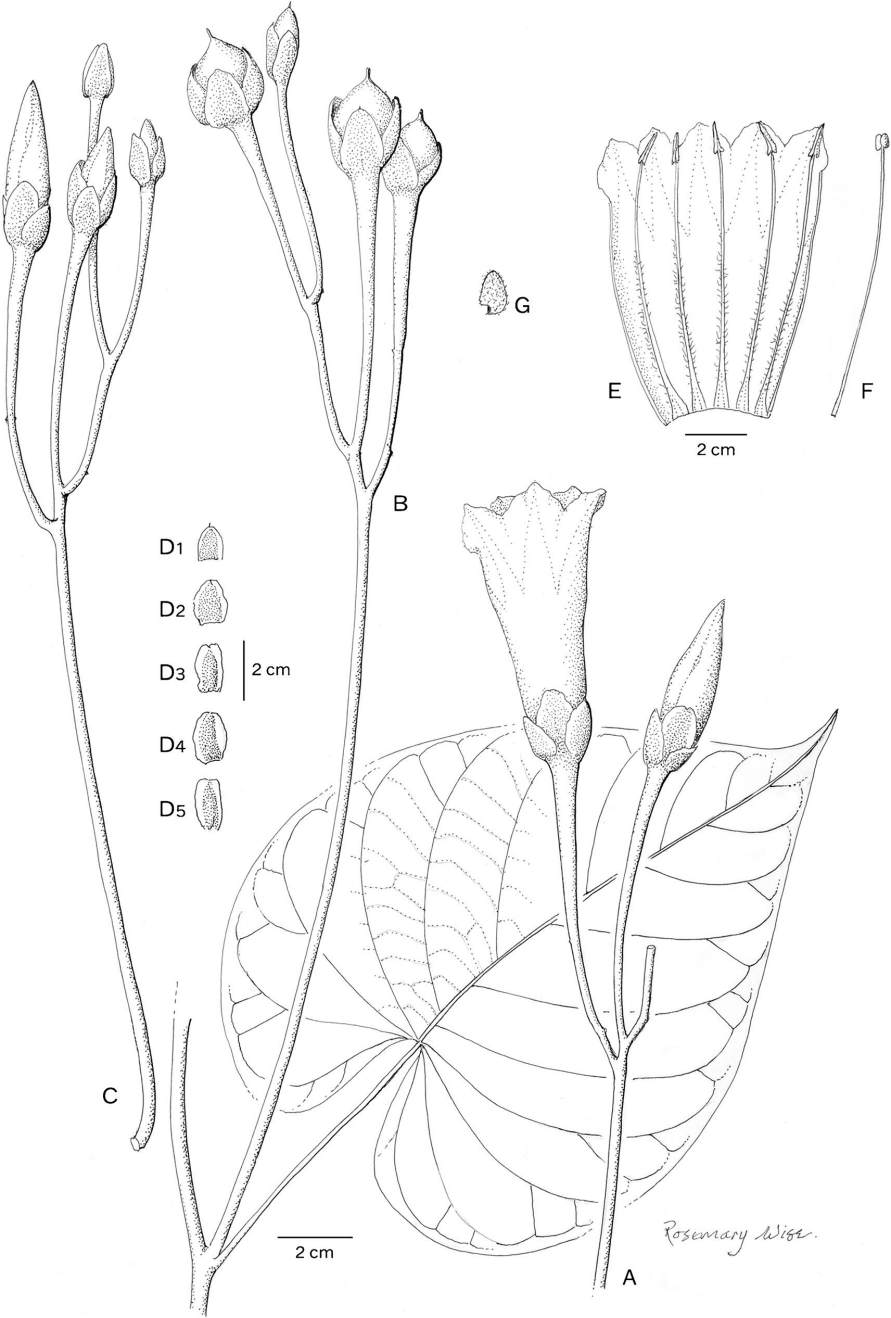
*Ipomoea ceronii* **A** flowering shoot; **B** fruiting inflorescence with leaf; **C** inflorescence with flowers in bud; **D** sepals, **1** outermost – **5** innermost; **E** corolla opened out to show stamens; **F** style; **G** seed. From *Wood et al.* 29619 and *Cerón & Reyes* 57501. drawn by rosemary wise.

**Fig. 4.**
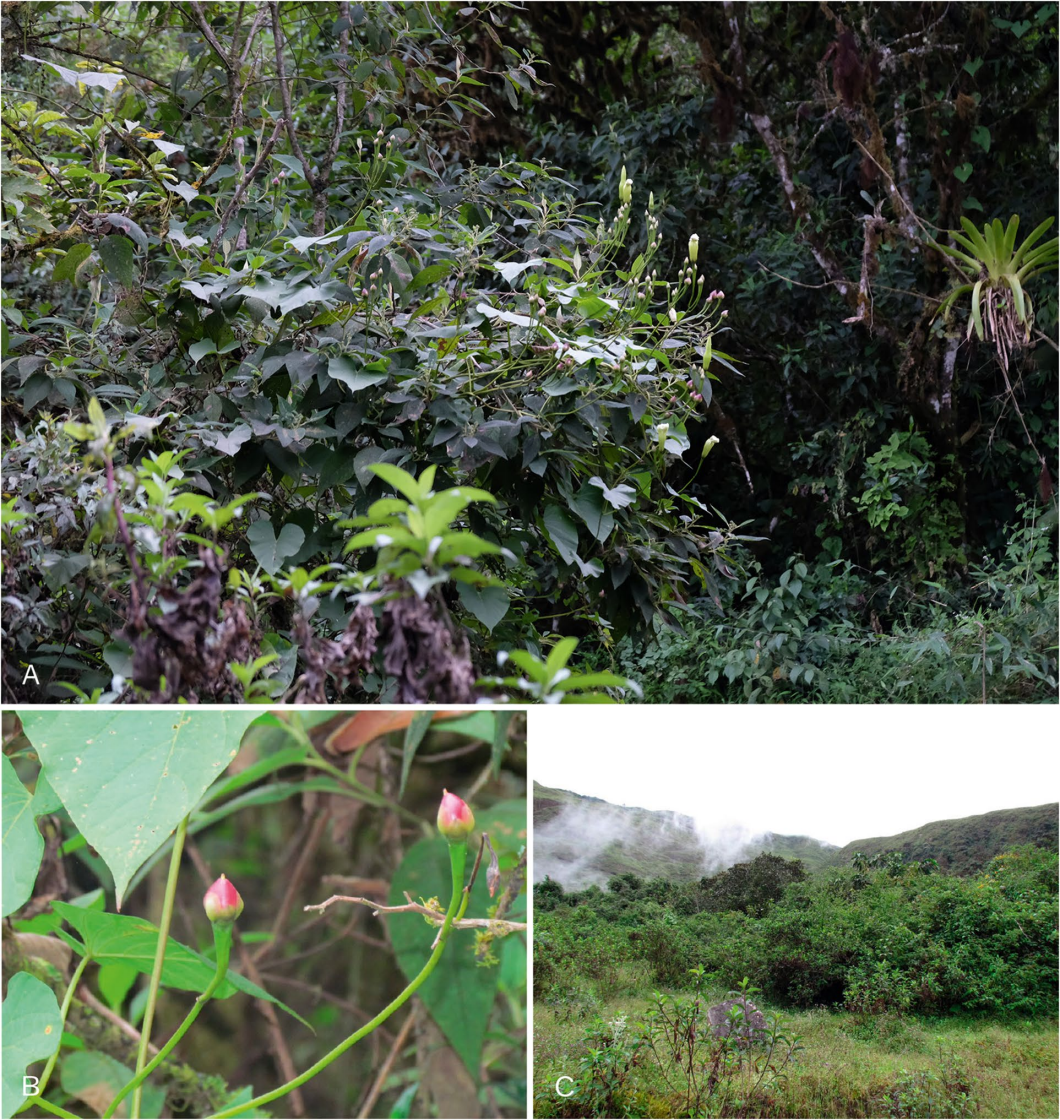
*Ipomoea ceronii* **A** habit and inflorescence; **B** fruit; **C** habitat. photos: pablo muñoz.

*Climbing perennial*; *stems* stout, glabrous. *Leaves* petiolate, 14 – 16 × 9.5 – 10.5 cm, ovate, shortly acuminate, cordate with rounded auricles, glabrous, abaxially slightly paler; petioles 4.5 – 8.5 cm, glabrous. *Inflorescence* of axillary cymes with 3 – 5 flowers; peduncles 23 – 38 cm long, stout, glabrous; bracteoles caducous, not seen; pedicels 2.5 – 7 cm long, the central slightly shorter than the laterals, notably thickened above. *Flowers* with sepals unequal; outer sepals 10 – 12 × 10 mm, triangular-narrowly ovate, obtuse, entirely green; inner sepals 14 – 15 × 5 mm, elliptic, obtuse to rounded; margins of both broad, scarious, transparent when fresh; corolla 7.5 – 8 cm long, tubular-funnel-shaped, but the tube only slightly widened from a broad base to c. 1.5 cm wide at mouth, externally greenish-white with greenish midpetaline bands but whitish internally, glabrous; limb pale yellow, shallowly lobed; filaments subequal, c.7.5 cm long, inserted at very base of corolla, basally 3 – 4 mm broad, pubescent, upwards narrower, glabrous; anthers linear, 8 – 9 × 1 mm, included; style 7 cm long, included, white, glabrous; stigma biglobose, brownish, 2 mm in diam. *Capsule* ovoid, shortly apiculate, 20 × 20 mm, becoming reddish upwards; *seeds* 19 × 12 mm, pubescent. Figs 3, 4A, B.

**recognition.** Molecular results, based on *Wood* 29619, show *Ipomoea ceronii* to be sister to the Argentinian species *I. jujuyensis* O’Donell in clade B (sensu Wood *et al.* 2020), discussed below under *I. velardei* O’Donell, with which it shares a relatively large apiculate capsule and scarious margined sepals but differs strikingly in its liana habit (not a perennial herb as in *I. jujuyensis*) and white (not pink) corolla, glabrous (not pubescent) stem and leaves and larger inner sepals up to 15 mm long (not up to 9 mm). *Ipomoea ceronii* is an entirely glabrous liana whose white flowers and oblong sepals recall the unrelated *I. reticulata* O’Donell but it is readily distinguished by the much larger corolla, c. 7.5 cm long (not 2.3 – 3.5 cm as in *I. reticulata*), unequal, broadly oblong sepals, 10 – 15 × 5 mm long (not subequal, elliptic, 5 – 7 × 3 – 5 mm) and extremely long peduncles to 24 cm in length (not to 4.5 cm) and pedicels 3.5 – 4 cm long (not 0.5 – 1.5) cm.

**Map 2.**
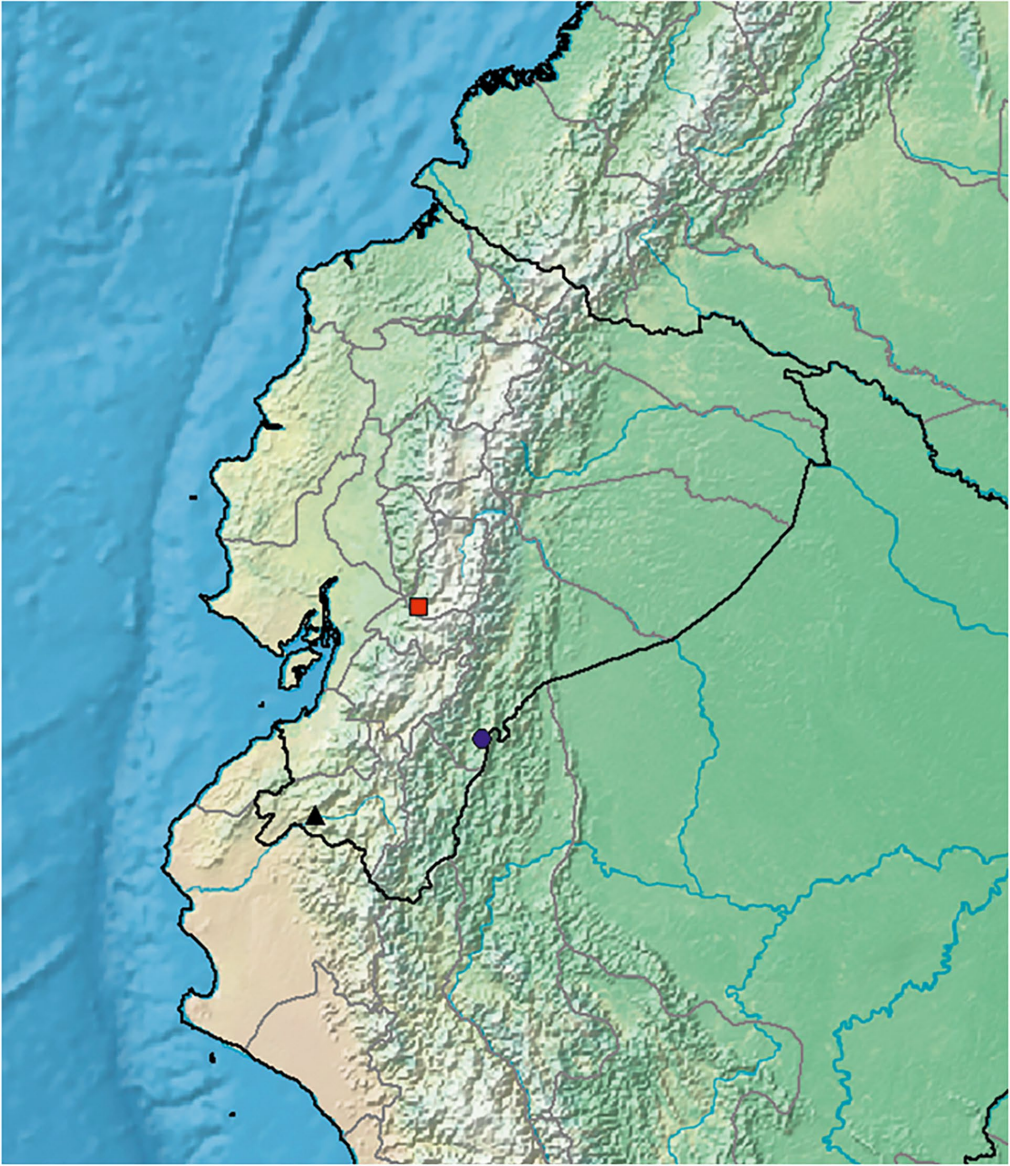
Distributions of *Ipomoea ceronii* (red square); *I. condorensis* (blue circle) and *I. papyrifera* (black triangle).

**distribution.** Endemic to a very small area of mountain forest above Huigra in Chimborazo Province of Andean Ecuador. Map 2.

**specimens examined. ecuador. Chimborazo:** Cantón Alausí, Parroquia Huigra, Sector Chasmay, 2°16'48"S 78°59'35"W, 1500 – 2000 m, common at 2000 m, 27 May 2006, *C. E. Cerón & C. Reyes* 57501 (QAP [QAP0060114]).

**habitat.** Evergreen humid broad-leaved mountain forest/cloud forest, 1500 – 2000 m.

**conservation status.** This species is restricted to an area of humid forest on steep mountain slopes above the town of Huigra in Chimborazo Province. Most of the forest has been cleared for pasture in the area and although the plant may cling on in other relict forest areas in the region there is no evidence for this. Given its very restricted range, and the vulnerability of its habitat to forest clearance, this species is probably Endangered [EN] according to IUCN guidelines (2012) but should provisionally be treated as Data Deficient [DD], until serious population studies have been carried out. Fig. 4C.

**etymology.** This species is named after Carlos E. Cerón Martínez, leading contemporary Ecuadorian botanist and original discoverer of this species. He has provided invaluable help to John Wood and colleagues.

**notes.** Neither field observations nor examination of the herbarium specimens enables exact description of the corolla. It appears to be essentially hypocrateriform but somewhat widened towards the apex. Although the filaments are nearly equal in length, the anthers are not exserted, as is usual in species with a hypocrateriform corolla, but held at the corolla mouth. The seeds are exceptionally large for *Ipomoea* reaching almost 2 cm in length.

**Ipomoea condorensis** *J.R.I.Wood & Scotland* **sp. nov.** Type: Ecuador, Zamora-Chinchipe: El Pangui - Región de la Cordillera del Condor (west side) - Río Quimi valley, 10 May 2007, *Wilson Quizhpe & A. Wisum* 2695 (holotype QCNE; isotypes LOJA (not seen), MO [MO-3148871], OXF).

http://www.ipni.org//urn:lsid:ipni.org:names:77348687-1

**Fig. 5.**
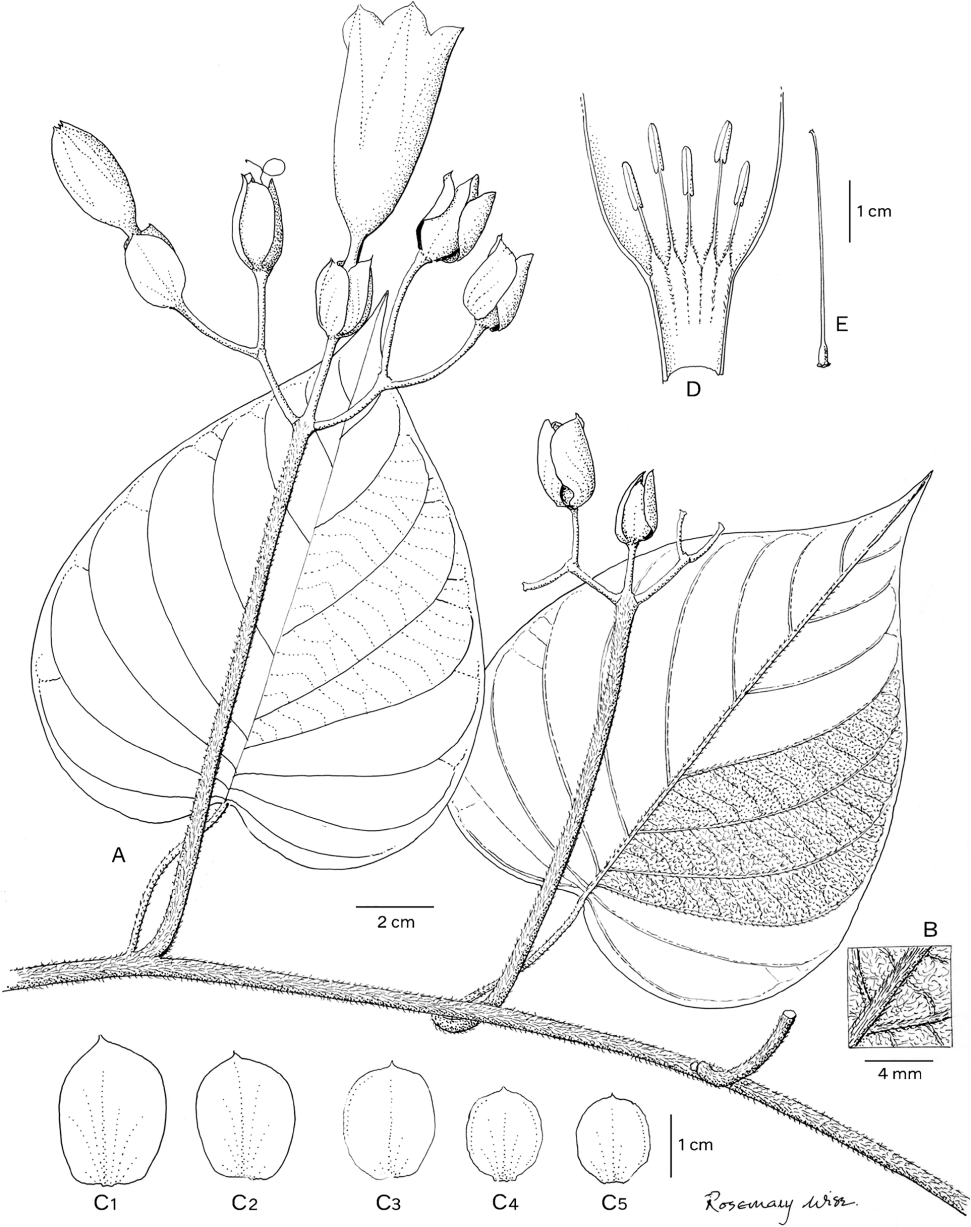
*Ipomoea condorensis* **A** habit; **B** abaxial surface of leaf; **C** sepals (**1** outermost – **5** innermost); **D** corolla opened out to show stamens; **E** ovary and style. From *Wilson Quizhpe & A. Wisum* 2695. drawn by rosemary wise.

*Liana* of unknown height; *stems* coffee-coloured, tomentose. *Leaves* petiolate; laminas 8 – 19 × 7.5 – 16 cm, ovate, cordate, apices finely acuminate, margins entire or shallowly lobed, adaxially dark green and glabrous, abaxially grey-tomentose; petioles 4 – 7 cm, tomentose. *Inflorescence* of axillary cymes; peduncle 9 – 19 cm, stout, brownish-tomentose; secondary peduncles 1.8 – 2.5 cm; bracteoles not seen; pedicels c. 1 cm, tomentellous with brownish hairs. *Flowers* with sepals subequal; sepals 20 – 23 × 10 – 12 mm, elliptic-obovate, obtuse to rounded, glabrous on exterior but inside shortly puberulent; corolla 6 – 7 cm long, shortly funnel-shaped above a cylindrical basal tube c. 1.5 cm long and 3 mm wide, white, glabrous; stamens unequal, glabrous except below; longer filaments c. 12 mm, shorter filaments c. 8 mm; style glabrous, c. 4.5 cm long, stigma 5-lobed, ovary glabrous. *Capsule* and *seeds* not seen. Fig. 5.

**recognition.** This species is distinctive because of the tomentose stems, leaves which are grey-tomentose abaxially and white corolla, superficially suggesting a tomentose, white-flowered form of *Ipomoea philomega* (Vell.) House but the large sepals, 20 – 23 mm long (not 11 – 16 mm as in *I. philomega*), which are internally pubescent (not glabrous), suggest a relationship with *I. magna* Sim.-Bianch. & J.R.I.Wood from Brazil. However, the corolla is white, not pink and the leaves are adaxially glabrous (not roughly tomentellous). It might also be compared with *I. nivea* J.R.I. Wood & Scotland from Peru, another liana with large sepals and tomentose stems, but differs in the leaves and sepals adaxially glabrous (not shortly tomentellous as in *I. nivea*) and especially by the much shorter, glabrous corolla, 6 – 7 cm long (not pubescent, c. 12 cm).

**distribution.** Endemic to Ecuador and only known from the type collection on the Cordillera del Condor which forms the boundary between Ecuador and Peru. Map 2.

**specimens examined. **Only known from the type.

**habitat.** Humid submontane forest, 900 m.

**conservation status.** Provisionally Data Deficient [DD] according to IUCN (2012) guidelines. There is no information about population size or threats to its habitat, but controversial mining activities are reported from the Cordillera del Condor.

**etymology.** This species is named after the Cordillera del Condor where it was found.

**notes.** There is a curious discrepancy in the labelling of the type collection. Although the collection number, coordinates and precise location are the same, the province and municipality of the MO and OXF isotypes are given as “Morona-Santiago, Cantón Gualaquiza”, whereas on the holotype they are given as “Zamora-Chinchipe: El Pangui”. We have cited the holotype location according to its label but are not in a position to confirm whether or not the location data is correct. It seems probable that the collectors were uncertain as to the exact province where the collection was made.

**Ipomoea ophiodes** *Standl. & Steyerm.* and **I. regnellii ***Meisn*.

**Fig. 6.**
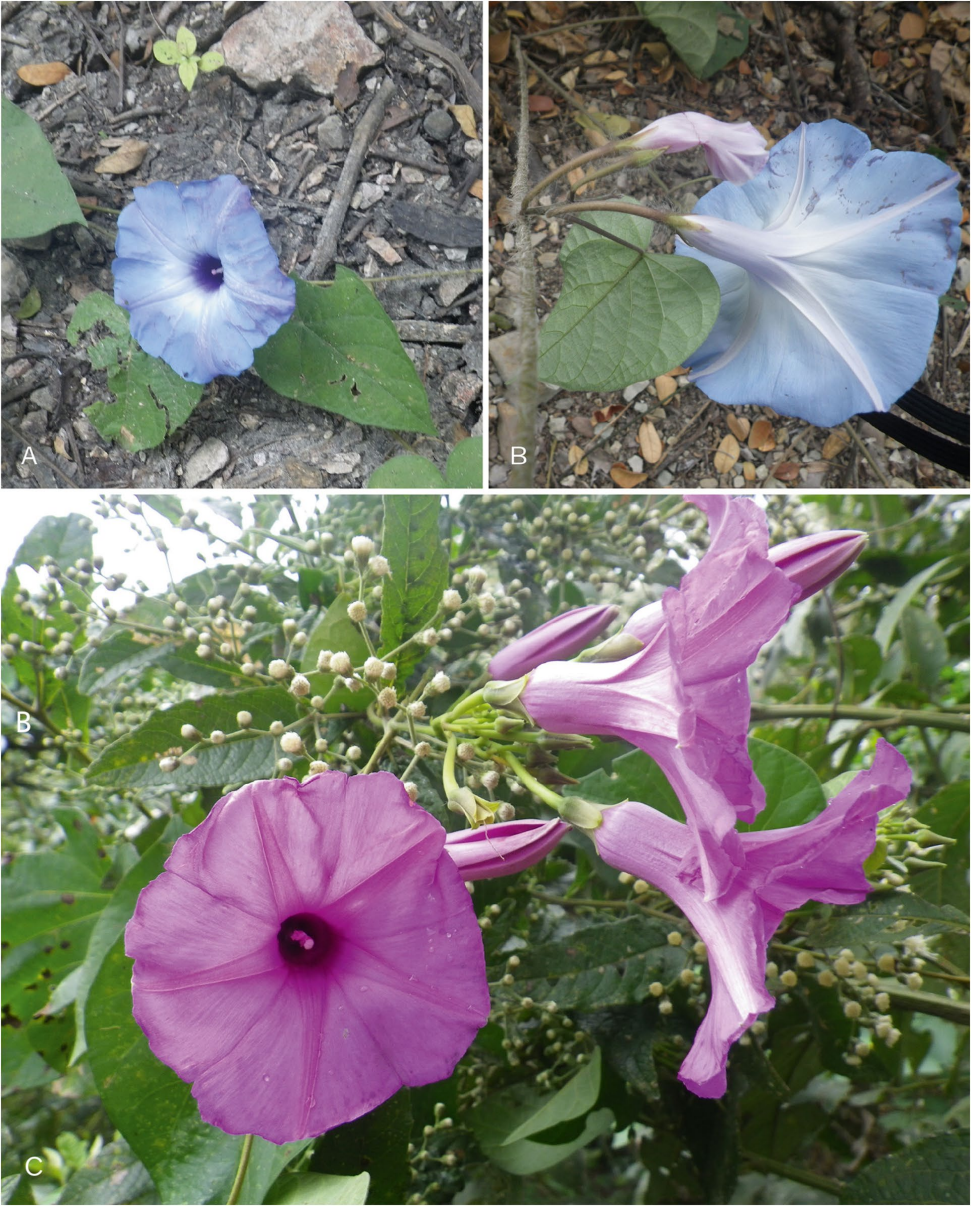
*Ipomoea ophiodes* **A** corolla; **B** sepals, note trailing pilose stem and corolla colour. *Ipomoea regnellii* **C** note corolla colour, sepal shape and climbing stem. photos: **A – B** john wood; **C** daniel soto.

In the monograph of *Ipomoea* (Wood *et al.* 2020), these two species were treated as conspecific, although a note was added about possibly distinct populations. After seeing extensive collections of plants named *I. ophiodes* in Ecuador and comparing them with populations of *I. regnellii*, it is obvious that the morphological distinctions between these two taxa are real. This was confirmed by our field observations and molecular results. Based on *J. R. I. Wood* 29610, molecular sequencing confirms that *I. ophiodes* is a distinct species in the Old World clade (sensu Wood *et al.* 2020). Although both species belong to the clade of species of American origin nested within the Old World Clade, *I. ophiodes* is part of a subclade of c. 11 species which includes *I. pedicellaris* Benth. and *I. corymbosa* (L.) Roth ex Roem. & Schult. On the other hand, *I. regnellii* forms another subclade with *I. chapadensis* J.R.I.Wood & L.V.Vasconc. and *I. chiquitensis* J.R.I.Wood & Scotland. Our results, therefore, show that both species belong to separate but related lineages and thus the name *I. ophiodes* should be resurrected (Fig. 1). The two species are illustrated in Fig. 6. *Ipomoea ophiodes* is commonly a trailing plant with pilose stems and sepals and 1 (– 2)-flowered cymes. The flower buds are glabrous and the corolla blue, although it fades to pink post-anthesis. In contrast, *I. regnellii* is a vigorous, scrambling or climbing plant whose stems and sepals are merely sparsely hirtellous and whose cymes are usually many-flowered. The flower buds are pubescent and the corolla pink.

**Ipomoea ophiodes** *Standl. & Steyerm*. (Standley & Steyermark 1944: 82). Type: Guatemala. Santa Rosa, Región de La Morenita, Dec. 1940, *P. C. Standley* 78884 (holotype F [F0054857]).

**specimens examined. ecuador.** **El Oro:** [Machala], entre Porvenir and Pénjamo, 80 m, *Linda Albert de Escobar* 1181 (QCA); ibid., 1182 (QCA). **Guayas:** Salinas-Guayaquil, 50 m, 2°13'S 80°23'W, *A. Barfod et al.* 48508 (QCA); 10 km N of Cerecita towards Julio Moreno, 50 m, 2°20'S 80°60'W, *G. Harling & L. Andersson* 25064 (QCA); sin data, *T. Núñez & E. Yagual* 466 (QCNE); Isla Puná, 2°48'S 80°00'W, 0 m, *Madsen* 63085 (QCNE, QCA); ibid., 2°45'S 79°55'W, 0 – 50 m, *J. E. Madsen* 63005 (QCA); ibid., *J. E. Madsen* 63613 (QCNE); ibid., 2°48'S 80°08'W, *J. E. Madsen* 63616 (QCA); Bosque Protector Cerro Blanco, 2°10'S 79°58'W, 40 m, *X. Cornejo* 205 (GUAY); ibid., *X. Cornejo* 206 (GUAY); Cerro Azul, Guayaquil, 25 March 1955, *E. Asplund* 15897 (K). **Loja:** 10 – 14 km E of Macara towards Cariamango, 550 m, 4°23'S 79°49'W, *J. E. Bohlin et al.* 1290 (GB, QCA); Bosque Petrificado Puyango, camino a Quebrada El Chirimoyo, 350 m, 3°53'S 80°04'W, *X & C. Cornejo* 4126 (GUAY); La Cruz, 500 m 4°22'31"S 79°58'57"W, 20 Sept 2000, *J. E. Madsen et al.* 7344 (LOJA); Hac. Banderones, El Limo-Casaderos Road, 1000 m, 3°58'66"S 80°10'715"W [80°10'43"W], 9 May 1997, *B. Klitgaard et al.* 535 (LOJA). **Los Ríos:** Est. Biol. Pedro Franco Dávila, 50 – 70 m, 1°14'S 79°38'W, *A. P. Yáñez & R. Foster* 189 (QCA); Río Palenque Field Station, km 56 Quevedo-Santo Domingo de los Colorados, 3°22'S 79°50'W, 200 m, *A. Gentry* 10155 (QCA); ibid., *C. H. Dodson & J. M. Vrieze* 4333 (QCA); ibid., *M. McMahon* 4260 (QCA). **Manabí:** Cantón Jipijapa, P. N. Machalilla, 2 – 5 km W of Guele, 1°41'S 80°46'W, 100 – 150 m, *A. P. Yañez et al.* 1311 (QCA, QCNE); Jipijapa – Pedro Carlos, 390 m, 1°21'S 80°35'W, *T. Plowman & P. Alcorn* 14365 (QCA); Los Camarones, entrada a Reserva Ecológica Jama-Coaque, 00°05'345"S [00°05'21"S] 80°09'315"W [80°09'19"W], 42 m, 29 May 2022, *J. R. I. Wood et al.* 29610 (QCA, OXF); c. 1 km N of Puerto López on road to Puerto Cayo, 1°32'1639"S [1°32'10"S] 80°47'58"W [80°47'35"W], 2700 m, 27 May 2022, *J. R. I. Wood et al.* 29618 (OXF, QCA).

**Map 3.**
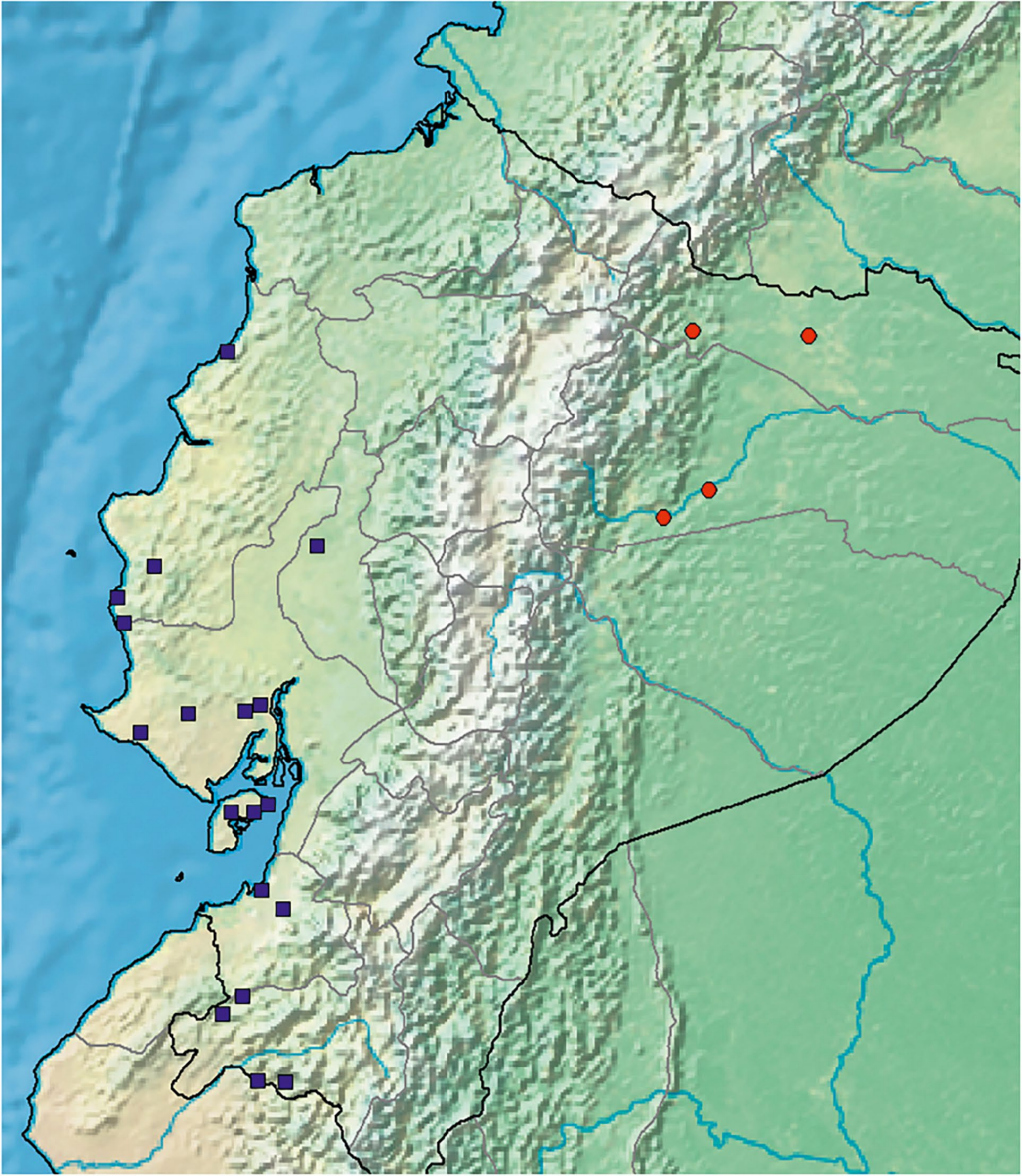
Distributions of *Ipomoea regnellii* (red circles) in the east of Ecuador and *I. ophiodes* (blue squares) in coastal Ecuador.

**notes.** The distinctive blue corolla and pilose stems suggest an intermediate position morphologically between *Ipomoea regnellii* and *I. clavata* (G.Don) Ooststr. ex J.F.Macbr. (Macbride 1931: 3), as these features are also characteristic of the latter species. Phylogenetic studies place all three species in a group of American species in the Old World Clade but not as sister species. Fig. 1.

**habitat & distribution.** In Ecuador frequent in dry forest in the departments along the Pacific coast: Manabí, Guayas, Los Ríos and Loja. Outside Ecuador it is recorded from similar habitats in Central America from Costa Rica, El Salvador, Guatemala and Honduras. Map 3.

**Ipomoea regnellii** *Meisn.* (Meisner 1869: 266). Type: Brazil, Minas Gerais, Caldas, s.d., *A. F. Regnell* s.n. (lectotype BR [BR00005793693], designated by O’Donell (1952) and redesignated as second step lectotype here to avoid possible ambiguity).

*Ipomoea warmingii* Meisn. (Meisner 1869: 272). Type: Brazil, Minas Gerais, *E. Warming* 1764 (holotype BR [BR00005793334]; isotype C).

**habitat & distribution.** A plant of humid forest and of scrub derived from moist forest in the Amazonian region of Ecuador. In South America, widespread in similar habitats around the Amazon basin from Colombia south to Bolivia and in similar habitats in central Brazil. (Wood *et al.* 2020: 738 – 740). Map 3.

**specimens examined. ecuador.** **Napo:** Cantón Tenam, Est. Biol. Jatún Sacha, 1°04'S 77°36'W, *B. C. Bennett et al.* 207-SFS (QCNE); entre Chonta Punto near Santa Rosa, 0°54'S 77°20'W, *H. Lugo* 2116 (QCA); Santa Rosa, Río Napo, 27 April 1972, *H. Lugo* 1963 (K); entre Río Napo and Río San Miguel, 300 m, 0°00'N, 76 – 77°W, *R. Navarrete* 24 (QCA); ibid., 39 (QCA). **Sucumbíos:** Río San Miguel, Puerto Nuevo, *G. Gutiérrez* 2679 (COL); Cantón Gonzalo Pizarro, 2 km S of Comuna Pandeyuca, Río Dashiño, 0°02'N, 77°26'W, *A. P. Yáñez et al.* 985 (QCA).

**Ipomoea papyrifera** *J.R.I.Wood & Scotland* **sp. nov.** Type: Ecuador, Loja: km 10 – 13, Macará – Zapotillo road, 500 – 600 m, 16 April 1980, *G. Harling & L. Andersson* 18334 (holotype QCA [QCA34457]; isotypes GB, MO [MO-5932377]).

http://www.ipni.org//urn:lsid:ipni.org:names:77348688-1

**Fig. 7.**
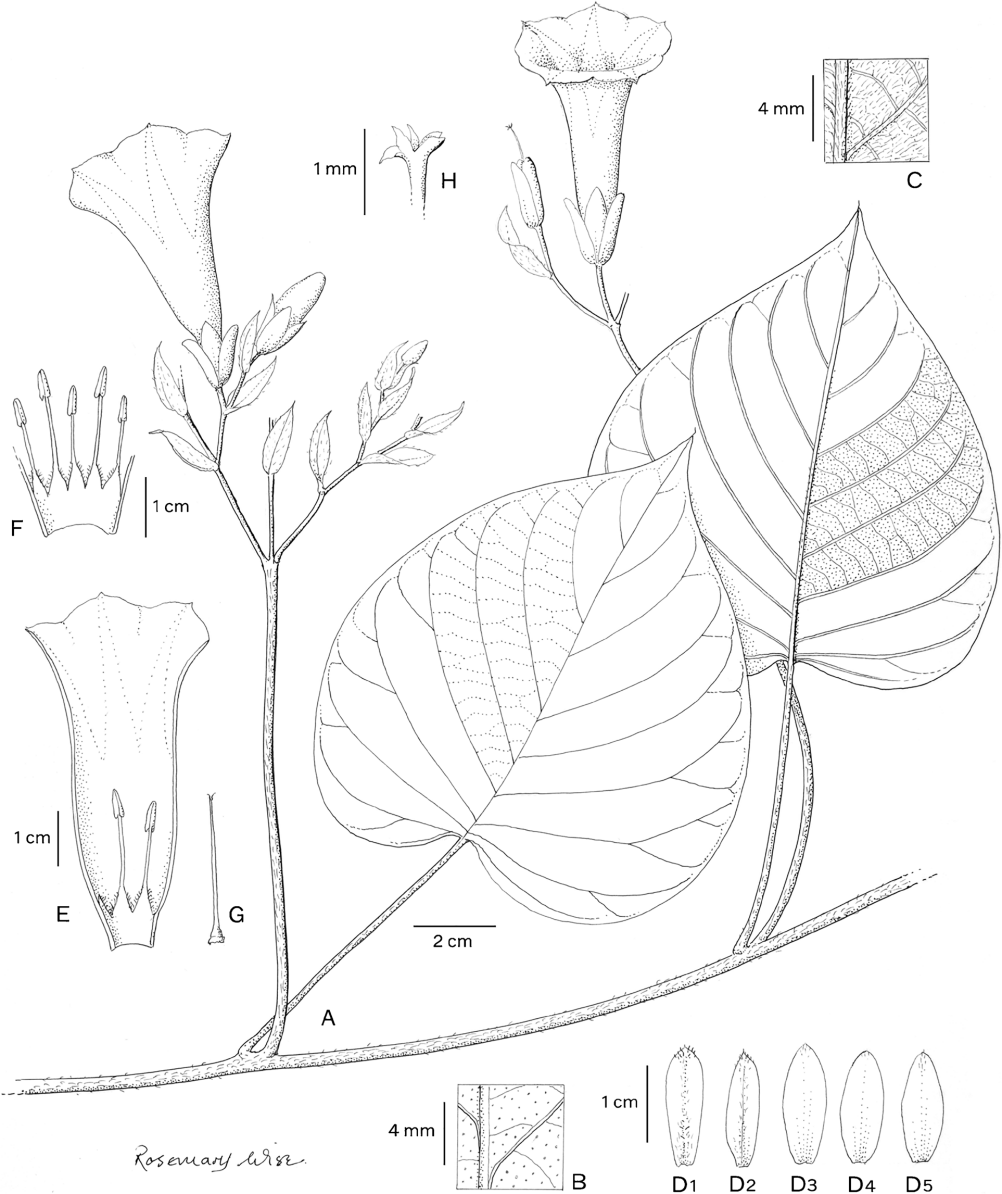
*Ipomoea papyrifera* **A** habit; **B** adaxial surface of leaf; **C** abaxial surface of leaf; **D** sepals (**1** outermost – **5** innermost); **E** half section of corolla; **F** stamens; **G** ovary and style; **H** stigma. From *G. Harling & L. Andersson* 18334. drawn by rosemary wise.

*Liana* of unknown height, scant latex present, *stems* pubescent, purplish. *Leaves* petiolate; laminas 10 – 10.5 × 8 – 9.5 cm, ovate, shallowly cordate to subtruncate, apices acute to shortly acuminate and very shortly mucronate, margins entire, adaxially glabrous and minutely scabrid with scattered hair bases, abaxially pubescent; petioles 6.5 – 9 cm, pubescent. *Inflorescence* of pedunculate, axillary compound cymes of up to 5 flowers; peduncles 2.5 – 9 cm, shortly pubescent; secondary peduncles 1.5 – 2 cm; tertiary and quaternary peduncles shorter; bracteoles 17 – 21 × 4 – 6 mm oblong-lanceolate, acute, persistent, papery, pubescent; pedicels 3 – 14 mm long. *Flowers* with sepals subequal, 18 – 20 × 6 – 7 mm, oblong, obtuse, papery, glabrous except comose apices and ciliolate, scarious margins; corolla 6.5 cm long, 2.5 cm wide at apex, narrowly funnel-shaped, reddish-violet, pilose; stamens unequal, glabrous except below; longer filaments c. 10 – 12 mm, shorter filaments 8 – 10 mm; style glabrous, stigma 5-lobed, ovary glabrous. *Capsule* and *seeds* not seen. Fig. 7.

**recognition.** Recalling *Ipomoea racemosa* Poir. from the Caribbean in the large, persistent, papery bracteoles but differing in the very lax, much branched inflorescence (not relatively compact, trifurcate as in *I. racemosa*), acuminate (not abruptly acute) bracteoles and larger corolla reaching 6.5 cm (not 5 cm) in length with included (not exserted) stamens.

**distribution.** Endemic to Ecuador and only known from the type collection. Map 2.

**specimens examined.** Only known from the type.

**habitat.** Low altitude, riverside scrub in deciduous forest, 500 – 600 m.

**conservation status.** Provisionally Data deficient [DD] according to IUCN (2012) guidelines. There is no information about population size or threats to its habitat.

**etymology.** The epithet *papyrifera* refers to the papery texture of the bracteoles.

**notes.** This is a puzzling species morphologically, resembling *Ipomoea racemosa* from Cuba and Hispaniola but we cannot confirm this relationship as we have not been able to sequence the plant successfully. The stigma appears to be 5-lobed, a very unusual character in *Ipomoea* but something similar is found in *I. decasperma* Hallier f. from Mexico and *I. longituba* Hallier f. from Madagascar.

**Ipomoea setosa** *Ker Gawl.* (Ker Gawler 1818: 335)

**Fig. 8.**
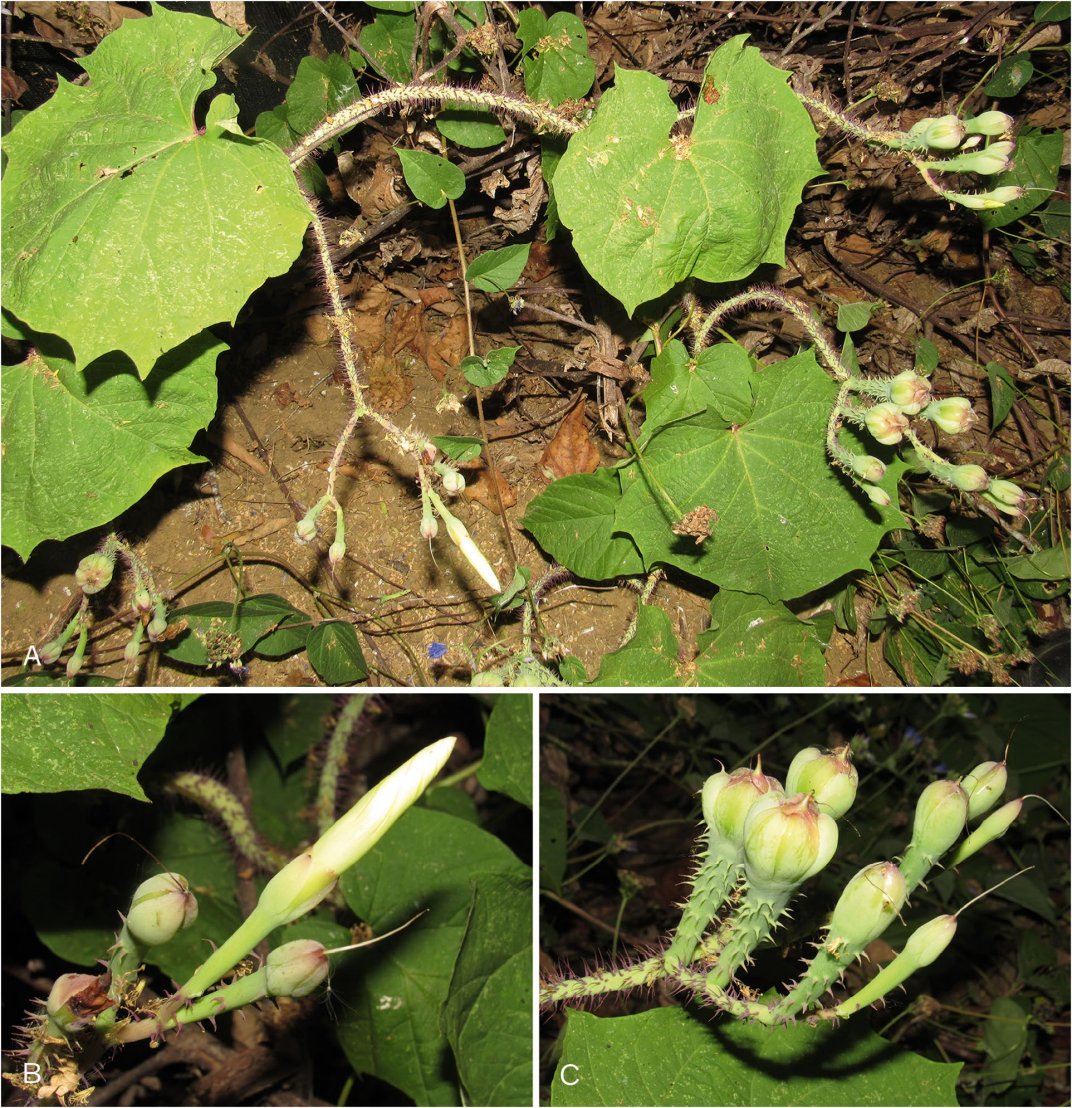
*Ipomoea setosa* subsp. *pavonii* (Hallier f.) J.R.I.Wood & Scotland, form with entire leaves **A** leaves; **B** flower; **C** fruit. photos: xavier cornejo.

Guayaquil is the type locality of *Ipomoea setosa* subsp. *pavonii* (Hallier f.) J.R.I.Wood & Scotland (Wood *et al.* 2020: 383), which is distinguished from the type subspecies by the absence of fleshy trichomes on the sepals, a relatively short corolla, 5 – 6.5 cm long, and 3-lobed leaves (Wood *et al.* 2020: 383ff.). Attention was drawn (*loc. cit.* 384) to an unusual form of subsp. *pavonii* present in northern Peru and characterised by its unlobed, coarsely dentate leaves. This form has now been found in Ecuador at Guayacán, west of Guayaquil, by Xavier Cornejo. Fig. 8 shows this form which may merit recognition after further study but it appears to differ only in the distinctive leaf shape.

**Ipomoea velardei** *O’Donell* and **I. jujuyensis** *O’Donell*

*Ipomoea jujuyensis* and *I. velardei* were both described in 1948 by O’Donell (1948a, b). *Ipomoea jujuyensis* is a relatively well-known species from Catamarca, Jujuy, Salta and Tucumán in Argentina. It is characterised by its pubescent, often lobed, leaves and axillary inflorescences with cymes of 1 – 5 pink flowers, the sepals pubescent but the corolla subglabrous, 6.5 – 9 cm long. The pubescence is variable in degree on all parts or completely absent. *Ipomoea velardei* was based on a specimen from the hills east of Lima in Peru. Superficially it is rather different, having a much smaller corolla, 2.5 – 4 cm long, pubescent on the exterior and somewhat narrower, with obtuse rather than rounded sepals. A handful of records of *I. velardei* are known from Peru (Wood *et al.* 2020), while *I. jujuyensis* has been collected only once in Peru near Cusco, but this perhaps requires confirmation as there is no recent record. Neither species is recorded from Bolivia.

Both species have been recorded from Ecuador, from where, in addition, *Ipomoea velardei* var. *aequatoriana* O’Donell was described by O’Donell (1952) based on a plant with a glabrous corolla. It should be noted that the observation that var. *aequatoriana* (Wood *et al.* 2020: 556) was sister to* I. meyeri* (Spreng.) G.Don was based on the molecular sequence of a misidentified specimen of var. *aequatoriana*. There is no reason to think that var. *aequatoriana* is a distinct species.

In the Loja herbarium we found three specimens with a small corolla and distinct inflorescence branching that fit *Ipomoea velardei* well. These are cited below. Two of the collections (*J. Armijas & F. Villena* 29; *B. Merino et al.* 4895) have a pubescent corolla whereas the third (*P. M. Jørgensen et al.* 1459) has a glabrous corolla conforming to var. *aequatoriana*.

**ecuador.** **Loja:** Paltas-Casanga, 1130 m, 31 May 2008, *J. Armijas & F. Villena* 29 (LOJA); Sabanilla, 4°11'56"S 80°07'08"W, 28 May 1996, *B. Merino et al.* 4895 (LOJA); Catamayo-Catacocha, km 25, turn-off at Las Chinchas towards Las Piñas, 3°57'21"S 79°29'07"W, 13 Dec. 1994, 2225 m, *P. M. Jørgensen et al.* 1459 (LOJA, MO, OXF).

**Fig. 9.**
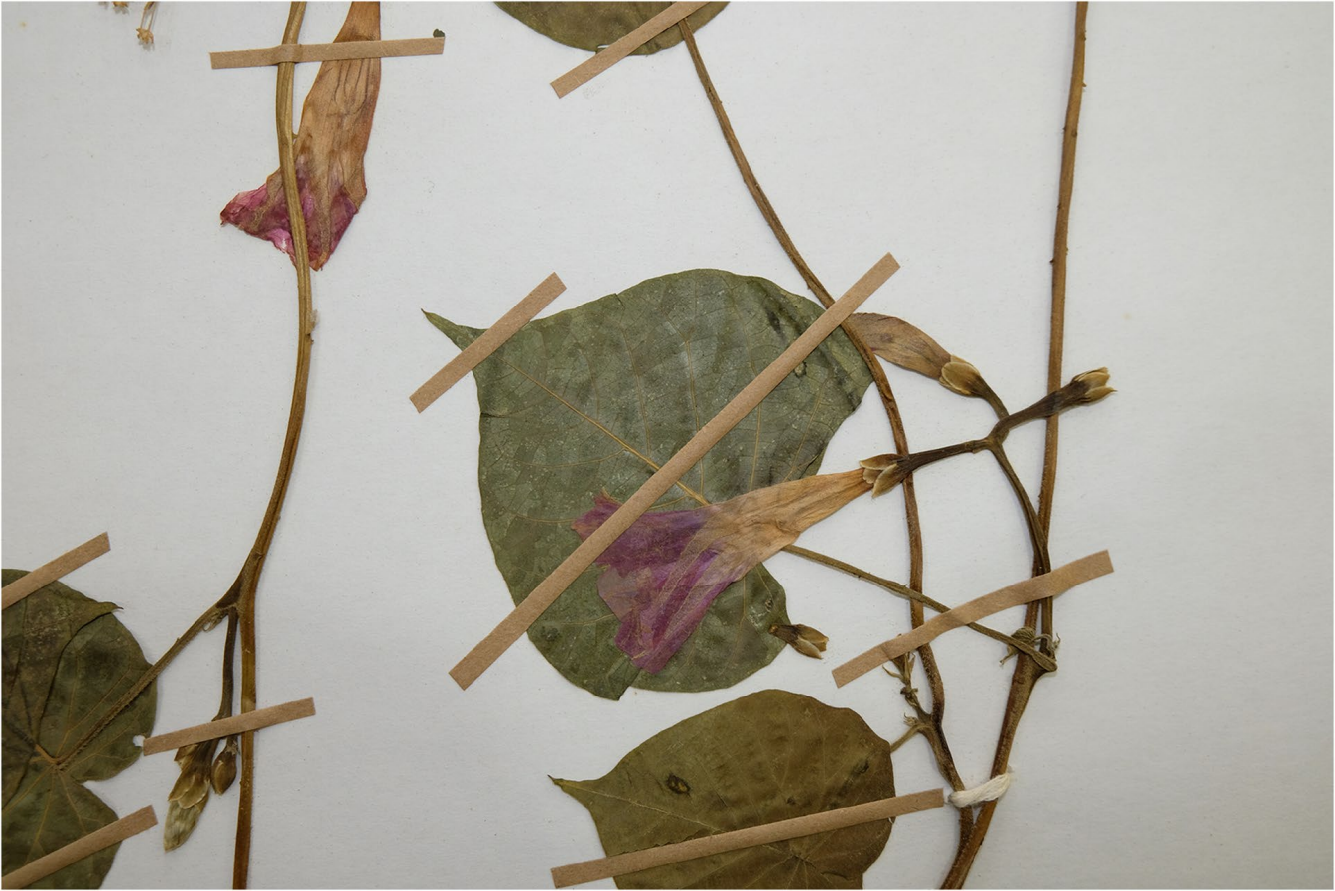
*Ipomoea velardei*. Herbarium specimen of *Merino et al.* 4895 (LOJA). Note distinctive inflorescence branching. photo: john wood.

Fig. 9 shows the rather distinctive inflorescence branching characteristic of *Ipomoea velardei.*

Around Loja, and particularly around Quito, there grows a more robust *Ipomoea* with a glabrous corolla which has been equated with *I. jujuyensis* (Austin 1982; Wood *et al.* 2020). These plants resemble *I. jujuyensis* very closely but it has always seemed unlikely that a plant with a distribution centred on the Jujuy region of Argentina would also occur in isolated populations near two cities in Ecuador. Populations in the Quito region, in particular, have an especially large glabrous corolla sometimes with a compound cymose inflorescence of 12 – 15 flowers, a number never attained by *I. jujuyensis* in Argentina. We, therefore, collected and sequenced samples of these plants and the results (Fig. 1) show this plant to be distinct from *I. jujuyensis* and apparently most closely related to *I. retropilosa* (Pittier) D.F.Austin from Colombia and Venezuela. This is described as a new species below and *I. jujuyensis* should be excluded from the list of species recorded from Ecuador.

**Ipomoea quitensis ***J.R.I.Wood & Cerón*** sp. nov.** Type: Ecuador, Pinchincha, Guápulo near Quito, 00°11'54"S 78°28'21"W, 25 May 2022, *J. R. I. Wood & C. E. Cerón Martínez* 29602 (holotype QCA; isotypes OXF, QAP).

http://www.ipni.org//urn:lsid:ipni.org:names:77348689-1

**Fig. 10.**
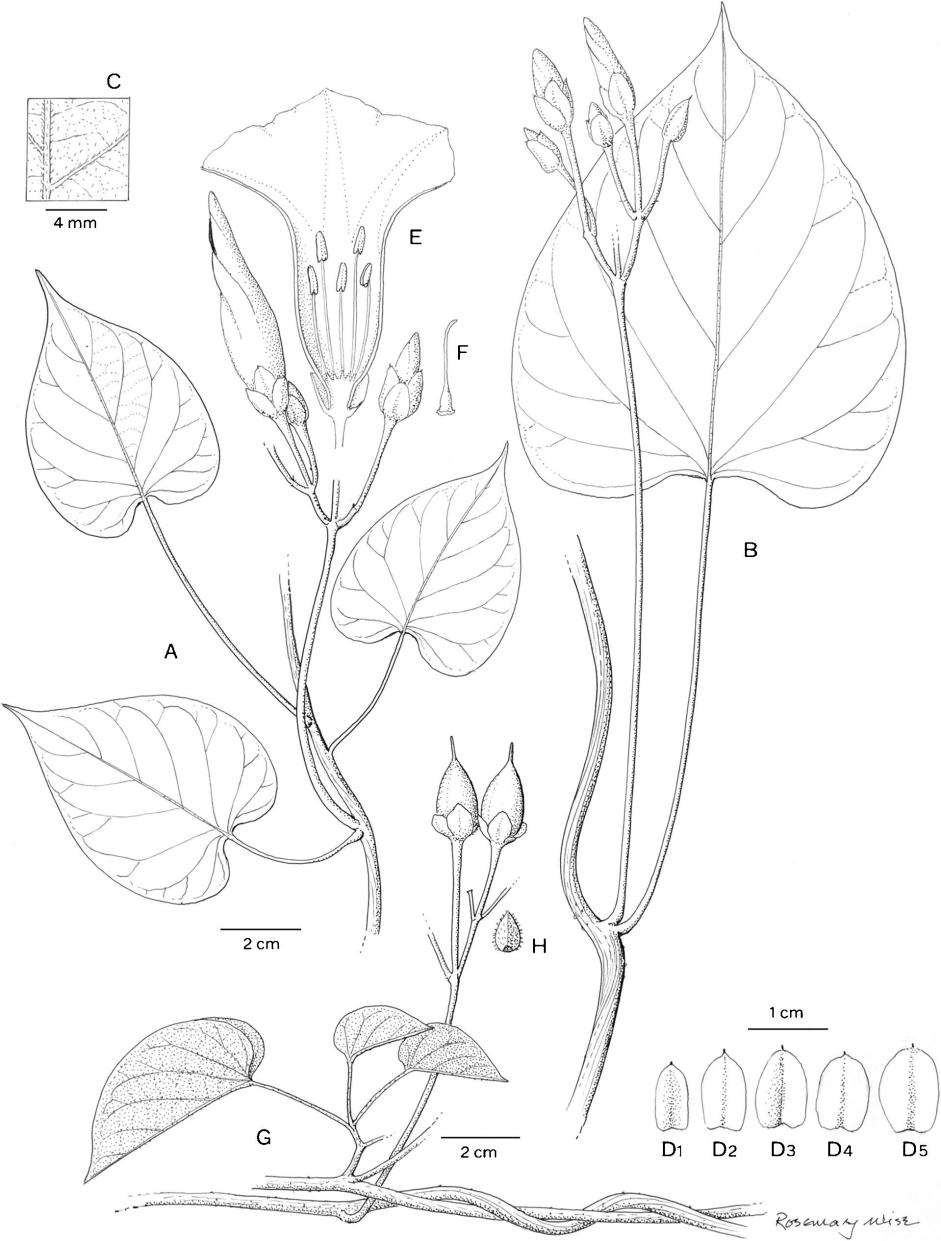
*Ipomoea quitensis*. **A** habit; **B** habit; **C** abaxial surface of leaf; **D** sepals (**1** outermost – **5** innermost); **E** corolla opened out to show stamens; **F** ovary and style; **G** fruiting branchlet with capsules; **H** seed.** A**, **C**, **E**, **F** from *Sparre* 14627; **B**, **D** from *Harling & Andersson* 14075; **G** – **H** from *Croat & Hannon* 92734. drawn by rosemary wise.

**Fig. 11.**
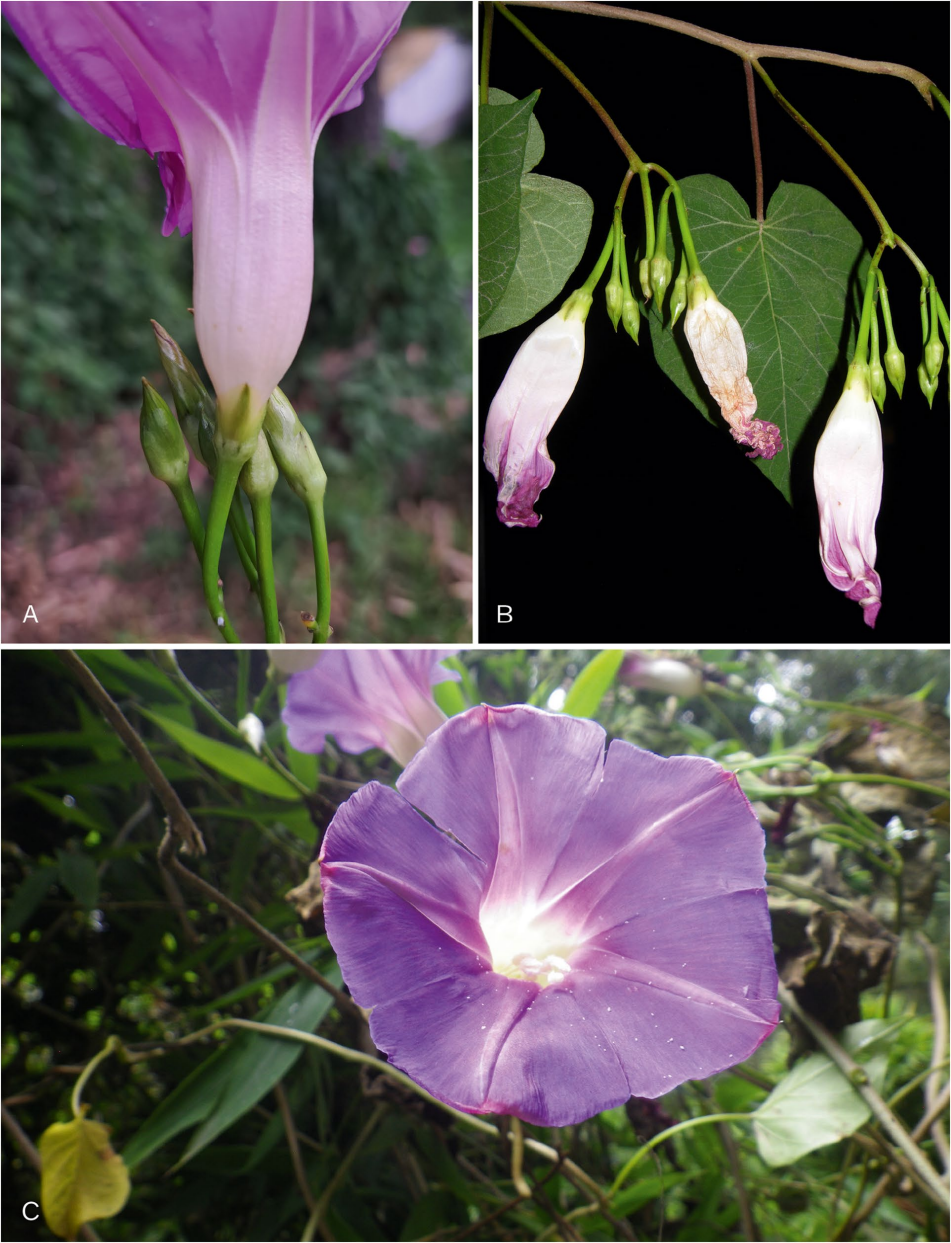
*Ipomoea quitensis*. **A** corolla and sepals; **B** inflorescence; **C** open corolla. photos: **a** david espinel; **b** carlos cerón; **c** john wood.

*Twining perennial*; *stem* pubescent with white based hairs, glabrescent and muricate when old. *Leaves* petiolate; 4.5 – 12 × 3 – 10 cm, ovate, broadly cordate, apices acuminate and minutely mucronate, margins entire to weakly undulate, adaxially pubescent, abaxially paler, densely pubescent; petioles 3 – 9 cm, pubescent. *Inflorescence* of compound axillary cymes with 3 – 14 flowers; peduncles 4 – 18 cm long; secondary peduncles 1 – 3.5 cm; tertiary peduncles 0. 5 – 2.5 cm or absent; bracteoles caducous, c. 2 mm long, lanceolate; pedicels 1.5 – 3.5 cm. *Flowers* with sepals slightly unequal, glabrous, the margins scarious; outer sepals ovate, obtuse to rounded, subterminally mucronate, 6 – 8 × 5 – 6 mm; inner sepals broadly elliptic, rounded, subterminally mucronate, 8 – 10 × 7 mm; corolla 7.5 – 8 cm long, funnel-shaped, abruptly widened above base, pink with paler tube, glabrous, mouth 4 – 5 cm in diam.; limb shallowly lobed; stamens unequal, glabrous except below; longer filaments c. 25 mm, shorter filaments 18 – 20 mm; style glabrous, stigma subglobose, exceeding anthers, ovary glabrous. *Capsule* ovoid, apiculate, 12 – 20 × 6 – 15 mm, glabrous; *seeds* ovoid, trigonous, 10 × 6 mm, hirsute. Figs 10, 11.

**recognition.** This species superficially resembles *Ipomoea jujuyensis* but is most easily distinguished by the many-flowered cymes with up to 14 flowers (not up to 5 as in *I. jujuyensis*) and the glabrous (not thinly pubescent) sepals. The leaves are always unlobed (those of *I. jujuyensis* are quite often lobed). Molecular studies, based on *J. R. I. Wood* 29602 and 29603 collected on the outskirts of Quito, show that *I. quitensis* belongs to a heterogeneous group of species in Clade B (sensu Wood *et al.* 2020) and is most closely related to *I. retropilosa* from Colombia and Venezuela. It differs in its funnel-shaped corolla with included stamens, whereas *I. retropilosa* has a hypocrateriform corolla with exserted stamens. Somewhat curiously, *I. retropilosa* subsp. *cundinamarcana* J.R.I.Wood & Scotland also shows a predilection for disturbed habitats near major urban centres, in its case Bogotá.

**Map 4.**
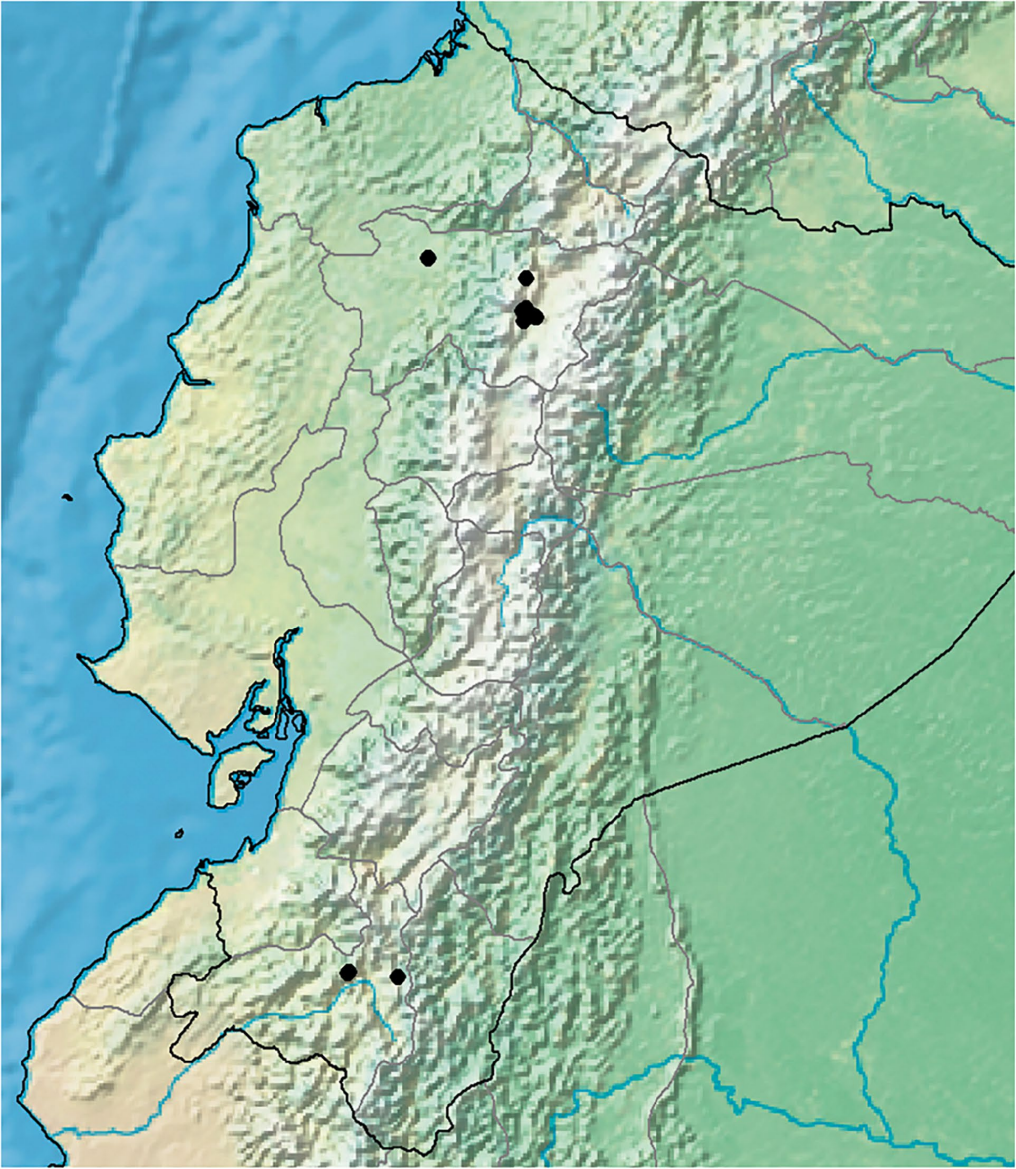
Distribution of *Ipomoea quitensis* near Quito and further south near Loja.

**distribution.** Endemic to Andean Ecuador, principally in and around the cities of Quito and Loja. The absence of records between these two centres is noteworthy and not readily explained. Map 4.

**specimens examined. ecuador.** **Pichincha:** Quito garden, April 1897, labelled “Ordo 145 Convolvulaceae”, *Sodiro* s.n. (Q); Quito, April 1897, *Sodiro* s.n. (Q); Parroquia Cumbayá, Cementerio municipal, 00°13'56"S 78°27'11"W, 2400 m, 25 Feb. 2018, *C. Cerón* 81797 (QAP); ibid., Quebrada de Guápulo, 2600 – 2800 m, 26 Feb. 1967, *B. Sparre* 14627 (AAU, S); Parque de Guápulo, 00°11'54"S 78°28'21"W, 2487 m, 24 May 2019, *C. Cerón* 85500 (QAP); Volcan Ilaló, entre Guangopolo and Tumbaco, camino central San Juan y Hac. Cunuyacu, 00°14'11"S 78°26'11"W to 00°14'51"S 78°25'10"W, 2500 – 2763 m, 11 April 2015, *C. Cerón et al.* 76028 (QAP); 10 km S of Tumbaco, lower slopes of Cerro Ilaló, 0°15'S 78°25'W, 2650 m, 7 April 1979, *L. Holm-Nielsen et al. *16976 (AAU, QCA); ibid., Parroquia Pomasqui, entre el complejo de Liga y la Avenida al pie del Cerro Catequilla. 00°06'28"S 78°29'21"W, 2600 m, *C. Cerón et al.* 73328 (QAP); ibid., Nayón, por el sector norte de Cumbayá, 2585 m, 0°13'S 78°30'W, 25 May 1997, *T. Romero* 0004 (QCA); Quito to Puerto Quito road, 10 km N of km 113, Reserva Forestal ENDESA, Río Silancha, Fundación Juan Manuel Durini 0°05'N 79°02'W, 650 – 700 m, 18 May 1987, *D. C. Daly et al.* 5203 (NY, QCA); exit from Quito on road pasando Mitad del Mundo vía Nanegalita, 00°01'485"S [00°01'29"S] 78°28'29"W [78°28'17"W], 2700 m, 27 May 2022, *J. R. I. Wood et al.* 29603 (OXF, QCA); cantón Rumiñahui, Parque Lineal Santa Clara, 0°19'3684"S [0°19'22"S] 78°26'368988"W [78°26'41"W], 2515 m, 30 Oct. 2023, *D. Espinel-Ortiz & C. Rodríguez* 386 (QCA). **Loja:** vic. Loja, 2300 m, 27 July 1939, *C. W. Penland & R. H. Summers* 1134 (GB, US); ibid., 2200 m, 15 April 1946, *R. Espinosa* 135 (LOJA); Loja – Zaruma, near Chinches, 2400 m, 30 April 1974, *G. Harling & L. Andersson* 14075 (AAU, GB, MO); Loja – Machala road, 3°57'39"S 79°28'39"W, 2317 m, *T. B. Croat & L. Hannon* 92734 (MO); Carretera Panamericana, entre Las Chinchas y Casatoma, 3.973883°N 79.49060°W, 2257 m, 6 June 2022, *P. Munoz-Rodriguez & D. Espinel* 51 (OXF, QCA).

**habitat.** Mountain scrub and disturbed bushy areas, (650 –) 2200 – 2763 m, such as remnants of Andean Forest and scrub including ‘matorral húmedo montano’, ‘matorral seco montano’, dry scrub, disturbed forest on undulating ground, quebradas, waste ground and disturbed bushy ground around gardens. It appears to be restricted to disturbed, secondary habitats. It sometimes occurs around gardens and may be cultivated in some cases.

**conservation status.** Although range restricted, this species seems to thrive in disturbed bushy habitats, the Parque Guápulo at the edge of Quito for example, and may in fact be spreading. Although detailed population assessments are desirable, it should be treated as provisionally Least Concern [LC] according to IUCN (2012) guidelines.

**etymology.** This species is named after the city of Quito, capital of Ecuador, as its distribution is centred on the city and its surroundings.

**notes.** This species has been identified in the *Flora of Ecuador* (Austin 1982) and generally in Ecuadorean herbaria as *Ipomoea jujuyensis*. Although the two species are superficially similar, this identification has always seemed improbable given the distribution of *I. jujuyensis*, which is a near endemic to Andean Argentina.

## Key to *Ipomoea* species occurring in Ecuador

The following key updates that found in the *Flora of Ecuador* (Austin 1982), particularly by the inclusion of the new species recognised and or discussed in this paper. Species not mentioned in the text above are: *I. abutiloides* G.Don; *I. acanthocarpa* (Choisy) Aschers. & Schweinf.; *I. alba* L.; *I. amnicola* Morong;* I*. *aquatica* Forssk.; *I. alexandrae* D.F.Austin; *I. aristolochiifolia* G.Don; *I*. *asarifolia* Roem. & Schult.; *I*. *capillacea* G.Don; *I. carnea* Jacq. subsp. *carnea; I. carnea* subsp. *fistulosa* (Mart. ex Choisy) D.F.Austin; *I*. *cairica* (L.) Sweet; *I. cholulensis* Kunth; *I*. *chondrosepala* Hallier f.; *I. chrysocalyx* D.F.Austin;* I*. *dubia* Roem. & Schult.;* I. dumetorum* Willd.; *I. habeliana* Oliv.; *I. harlingii* D.F.Austin;* I*. *hederifolia* L.; *I*. *heptaphylla* Sweet; *I*. *imperati* (Vahl) Griseb.; *I. incarnata* Choisy; *I. indica* (Burm.) Merr.; *I. jalapa* (L.) Pursh;* I*. *lactifera* J.R.I.Wood & Scotland; *I. leucantha* Jacq.; *I. mauritiana* Jacq.; *I*. *mucronata* Schery; *I. nationis* (Hook.) G.Nicolson; *I. nil* (L.) Roth;* I*. *pauciflora* M.Martens & Galeotti;* I*. *pes-caprae* (L.) R.Br.; *I. purpurea* (L.) Roth; *I. quamoclit* L.; *I. ramosissima* Choisy; *I. rubens* Choisy; *I. setifera* Poir.;* I*. *squamosa* Choisy; *I. tiliifolia* (Desr.) Roem. & Schult.;* I*. *tricolor* Cav.; *I. triloba* L. and *I*. *wolcottiana* Rose.

- 1. Leaves pinnate .................................................................................................................. ...................................................................................................**I. quamoclit**
- Leaves entire or palmately lobed ..................................................................................................................................................................................................................................................................................................... 2
- 2. Corolla salver-shaped, white, the cylindrical tube 7 – 15 cm long .................................................................................................................................................... 3
- Corolla variously shaped but if salver-shaped neither white nor with a cylindrical tube 7 – 15 cm long .................................................................................................................................................... 4
- 3. Liana with lanceolate to ovate leaves, cuneate at the base, much longer than broad; sepals truncate (Galapagos Islands) .................................................................................................................................................... ...............................................................................................................................................**I. habeliana**
- Vigorous twining herb with ovate, cordate leaves, nearly as broad as long; sepals awned ....................................................................................................................................................**I. alba**
- 4. Stems, peduncles and pedicels densely covered with stiff, somewhat fleshy, straight trichomes .................................................................................................................................................... 5
- Stems, peduncles and pedicels without stiff, straight somewhat fleshy trichomes; occasionally scattered, bent, fleshy spines present on stems only .....................................................................................................................................................6
- 5. Trichomes on sepals as well as stem; leaves usually 5-lobed ............................................................................................................................................................................................................. **I. setosa** subsp. **setosa**
- Trichomes restricted to the stems, peduncles and pedicels, but absent or nearly absent from the sepals; leaves 3-lobed or unlobed ............................................................................................................................................................................................................ **I. setosa** subsp. **pavonii**
- 6. Sepals with a distinct terminal or subterminal awn; corolla salver-shaped ....................................................................................................................................... 7
- Sepals lacking a distinct terminal or subterminal awn; corolla not salver-shaped (except *I. alexandrae*) ..................................................................................................................................... 10
- 7. Night-flowering species with dull, bluish flower; awn terminal; stems often with soft, fleshy teeth ................................................................................................................................ **I. mucronata**
- Day flowering species with red flowers; awn subterminal; stems lacking fleshy teeth ................................................................................................................................................... 8
- 8. Inner sepals short, usually < 3 mm long; capsule muticous; leaves commonly lobed .................................................................................................................................................... .................................................................................................**I. hederifolia**
- Inner sepals 4 – 6 mm; capsule rostrate; leaves entire .............................................................................................................................................. 9
- 9. Ovary and capsule usually pubescent; sepal awns 4 – 8 mm; fruiting capsule erect .................................................................................................................................................... . **I. dubia**
- Ovary and capsule glabrous; sepal awns 2 – 3.5 mm long; fruiting capsule usually recurved ....................................................................................................................................... **I. cholulensis**
- 10. Leaves palmately divided into separate segments ....................................................................................................................................... 11
- Leaves entire or, if lobed, not divided to base ....................................................................................................................................... 13
- 11. Small, usually erect, Andean species with corm-like rootstock; sepals muricate .................................................................................................................................................... **I. capillacea**
- Twining herbs lacking corm-like rootstock; sepals smooth ....................................................................................................................................... 12
- 12. Annual herb, the corolla < 2.2 cm long; peduncles usually twisted or coiled; pseudo-stipules absent ....................................................................................................................................... **I. heptaphylla**
- Perennial herb with corolla > 4 cm long; peduncles straight; pseudo-stipules present .................................................................................................................................................... **I. cairica**
- 13. Erect shrubs or treelets ....................................................................................................................................... 14
- Twining, scrambling or trailing herbs or lianas ....................................................................................................................................... 16
- 14. Corolla pink; sepals 5 – 6 mm long; leaves, sepals and corolla conspicuously pubescent .......................................................................................................................................**I. carnea** subsp. **fistulosa**
- Corolla white, often with dark throat; sepals 5.5 – 13 mm long; leaves, sepals and corolla glabrous or inconspicuously pubescent ....................................................................................................................................... 15
- 15. Plant completely glabrous, growing at 1000 – 2600 m .......................................................................................................................................**I. pauciflora**
- Sepals and leaves abaxially pubescent, growing below 900 m ....................................................................................................................................... **I. wolcottiana**
- 16. Seaside plants growing on shoreline or near brackish inlets; trailing glabrous plants rooting at the nodes and with leaf tips truncate or emarginate ....................................................................................................................................... 17
- Plants not growing by the sea but, if so, climbing, hirsute and or with acute leaf tips....................................................................................................................................... 19
- 17. Leaves broadly or narrowly oblong; corolla white with yellow throat ....................................................................................................................................... **I. imperati**
- Leaves ovate-cordate or subreniform; corolla pink or (rarely) pure white ....................................................................................................................................... 18
- 18. Leaves ovate, cordate, emarginate; sepals slightly unequal, smooth (usually on sea shores) ....................................................................................................................................... **I. pes-caprae**
- Leaves subreniform with truncate base and rounded apex; sepals very unequal in size, often muricate (usually but not strictly maritime) ....................................................................................................................................... **I. asarifolia**
- 19. Corolla salver-shaped, red; stamens exserted ....................................................................................................................................... **I. alexandrae**
- Corolla funnel-shaped or campanulate, variously coloured but not red, stamens included .......................................................................................................................................20
- 20. Inflorescence a pedunculate, subcapitate cluster, the bracteoles persistent, grey-tomentose ....................................................................................................................................... **I. amazonica**
- Inflorescence clearly cymose, usually lax, but if subcapitate, bracteoles caducous and/or not grey-tomentose .......................................................................................................................................21
- 21. Corolla pubescent or pilose on the exterior (check buds); sepals usually pubescent .......................................................................................................................................22
- Corolla glabrous on the exterior, even in bud; sepals glabrous or hairy ....................................................................................................................................... 31
- 22. Sepals linear-oblong, 3 – 4 mm wide, much longer than broad ....................................................................................................................................... **I. regnellii**
- Sepals oblong, ovate, elliptic or suborbicular, at least 4 mm wide ....................................................................................................................................... 23
- 23. Stems, leaves and sepals with spreading yellowish hairs....................................................................................................................................... **I. harlingii**
- Stems, leaves and sepals lacking spreading, yellowish hairs.......................................................................................................................................24
- 24. Sepals 5 – 6 mm long, very small relative to corolla ..............................................................................................................................................................................................................**I. carnea** subsp. **carnea**
- Sepals > 7 mm long ....................................................................................................................................... 25
- 25. Corolla large, 7 – 13 cm long ....................................................................................................................................... **I. jalapa**
- Corolla 3 – 7 cm long .......................................................................................................................................26
- 26. Sepals strongly accrescent in fruit and enclosing capsule; leaves with black gland dots on abaxial surface; coastal plant of the Galapagos Islands ....................................................................................................................................... **I. tiliifolia**
- Sepals not strikingly accrescent, nor enclosing the capsule; leaves without gland dots on abaxial surface; plants not usually coastal ....................................................................................................................................... 27
- 27. Bracteoles 17 – 21 mm long, persistent, oblong-lanceolate; sepals 18 – 20 mm long ....................................................................................................................................... **I. papyrifera**
- Bracteoles 7 – 15 mm long, persistent or caducous, usually linear, sepals c. 14 mm long .......................................................................................................................................28
- 28. Liana, reaching 7 m; sepals rounded to emarginate ....................................................................................................................................... **I. abutiloides**
- Herb, rarely exceeding 2 m; sepals obtuse to acute ....................................................................................................................................... 29
- 29. Sepals conspicuously pubescent to tomentose, > 10 mm long ....................................................................................................................................... 30
- Sepals ± glabrous, < 10 mm long ....................................................................................................................................... **I. velardei**
- 30. Pedicels very short, < 5 mm long; sepals oblong-ovate....................................................................................................................................... **I. chrysocalyx**
- Pedicels 5 – 17 mm long; sepals ovate-deltoid ....................................................................................................................................... **I. rubens**
- 31. Outer sepals with 3 – 5 prominent raised veins; bracteoles prominent, persistent.......................................................................................................................................**I. setifera**
- Outer sepals lacking prominent raised veins ................................................................................................................................................... 32
- 32. Corolla small, < 3 cm long ................................................................................................................................................... 33
- Corolla larger, > 3 cm long, often much larger ............................................................................................................................................. 42
- 33. Corolla cream, sometimes with dark centre; liana............................................................................................................................................... 34
- Corolla pink or blue (rarely white); annual or perennial herbs .............................................................................................................................................. 35
- 34. Sepals 10 – 14 mm long, oblong, spreading in fruit .......................................................................................................................................**I. corymbosa**
- Sepals 5 – 7 mm long, elliptic, remaining erect in fruit ....................................................................................................................................... **I. reticulata**
- 35. Perennial herb with slightly succulent leaves; sepals smooth, glabrous ....................................................................................................................................... **I. amnicola**
- Slender, usually annual herbs; leaves not succulent; sepals glabrous or hirsute, smooth or with warty excrescences ....................................................................................................................................... 36
- 36. Inflorescence usually dense, the pedicels short; sepals with spreading hairs; bracteoles persistent; corolla blue................................................................................................................................................ **I. meyeri**
- Inflorescence lax or dense, the pedicels variable in length; corolla pink or white, but, if blue, sepals glabrous .............................................................................................................................................. 37
- 37. Sepals terminating in a fine mucro, glabrous or hirsute, lacking whitish margins .............................................................................................................................................. 38
- Sepals acute or obtuse but not terminating on a fine mucro, glabrous, the margin pale, whitish .................................................................................................................................................... 40
- 38. Outer sepals elliptic-obovate, 1-veined; ovary and capsule glabrous ....................................................................................................................................... **I. ramosissima**
- Outer sepals lanceolate or oblong-lanceolate, 3 – 5-veined; ovary and capsule hirsute............................................................................................................................................. 39
- 39. Sepals oblong, 5 – 6 mm long ............................................................................................................................................... **I. triloba**
- Sepals oblong-lanceolate, 10 – 14 mm long ...................................................................................................................................................................................................................................................... **I. leucantha**
- 40. Peduncle passing through leaf sinus; corolla blue with white tube ....................................................................................................................................... **I. aristolochiifolia**
- Peduncle not passing through leaf sinus; corolla pink, rarely white ................................................................................................................................................ 41
- 41. Sepals with distinct dark blotches; pedicels mostly > 10 mm long; seeds puberulent ....................................................................................................................................... **I. dumetorum**
- Sepals lacking dark blotches; pedicels usually < 5 mm long; seeds pilose .................................................................................................................................................... **I. acanthocarpa**
- 42. Sepals with prominent spreading hairs, especially towards their bases ............................................................................................................................................. 43
- Sepals glabrous or shortly pubescent, prominent spreading hairs absent ............................................................................................................................................... 45
- 43. Flowers usually solitary; sepals narrowly lanceolate, c. 10 – 11 × 1.5 – 2 mm .......................................................................................................................................**I. ophiodes**
- Flowers usually several in compact cymes; sepals varied in shape but 11 – 32 × 2 – 5 mm ............................................................................................................................................. 44
- 44. Sepals 15 – 32 mm long, tapering to a long linear point; corolla blue when fresh; leaves 3-lobed .......................................................................................................................................**I. nil**
- Sepals 11 – 17 mm long, shortly acuminate to obtuse; corolla usually pink; leaves entire or (less commonly) 3 – 5-lobed ....................................................................................................................................... **I. purpurea**
- 45. Sepals large, 17 – 28 mm long, glabrous on the exterior .................................................................................................................................................... ..............................................................................................................................................46
- Sepals smaller, < 17 mm long, but if > 17 mm long, pubescent on the exterior .............................................................................................................................................. 48
- 46. Corolla white; stems tomentose; sepals elliptic to obovate, puberulent inside ....................................................................................................................................... **I. condorensis**
- Corolla pink or blue; stems glabrous or pilose; sepals lanceolate, glabrous on both surfaces ................................................................................................................................................47
- 47. Sepals prominently veined; stems glabrous; corolla pink .......................................................................................................................................**I. incarnata**
- Sepals not prominently veined; stems usually with long, white, spreading hairs; corolla blue .......................................................................................................................................**I. clavata**
- 48. Leaves palmately lobed; sepals coriaceous, rounded .......................................................................................................................................**I. mauritiana**
- Leaves entire or lobed; sepals varied in texture, but if coriaceous leaves entire .................................................................................................................................................. 49
- 49. Flowers clustered at apex of peduncle, the pedicels very short; leaves lobed or, less commonly entire; sepals usually pubescent or hirsute .......................................................................................................................................50
- Flowers not clustered, the pedicels of varying lengths, leaves entire, sepals usually glabrous ...............................................................................................................................................52
- 50. Bracteoles persistent; stigma 3-lobed; twining herb with corolla 5 – 6 cm long ....................................................................................................................................... **I. indica**
- Bracteoles caducous; stigma bilobed; prostrate, ascending or twining herb with corolla 4 – 4.5 cm long .......................................................................................................................................51
- 51. Outer sepals < 7 mm long; inner sepals < 10 mm; twining plant with stems 1 – 3 mm diam. and internodes 6 – 16 cm .......................................................................................................................................**I. aequatoriensis**
- Outer sepals > 7 mm long, inner sepals > 12 mm long; trailing or ascending herb with stems 2 – 6 mm diam. and internodes 2 – 10 cm .......................................................................................................................................**I. batatas**
- 52. Completely glabrous, trailing, procumbent or floating herbs .............................................................................................................................................. 53
- Twining herbs, glabrous or hirsute ...............................................................................................................................................54
- 53. Trailing herb with subreniform leaves; sepals unequal, the outer often muricate abaxially .......................................................................................................................................**I. asarifolia**
- Aquatic herb, free-floating on water or procumbent and rooting on mud; sepals subequal, smooth abaxially .......................................................................................................................................**I. aquatica**
- 54. Corolla cream or yellow-green, sometimes with a dark centre; sepals obtuse or rounded ...............................................................................................................................................55
- Corolla pink, but if white, sepals mucronate .................................................................................................................................................... 57
- 55. Sepals < 10 mm long; peduncles < 10 cm long; pedicels 0.5 – 1.5 mm long; capsule up to 1 cm long; leaves often gland-dotted beneath ..............................................................................................................................................56
- Sepals 10 – 15 mm long; peduncles 23 – 38 cm long; pedicels 2.5 – 7 cm long; capsule c. 2 cm long; leaves not gland-dotted beneath .......................................................................................................................................**I. ceronii**
- 56. Corolla 2.5 – 3.5 cm long, ± campanulate; inflorescence often compounded and subracemose or ± paniculate .............................................................................................................................................. **I. reticulata**
- Corolla 4 – 8 cm long, funnel-shaped; inflorescence clearly cymose ....................................................................................................................................... **I. batatoides**
- 57. Sepals conspicuously mucronate; corolla 3 – 4.5 cm long .............................................................................................................................................. 58
- Sepals rounded, obtuse or acute but not obviously mucronate; corolla up to 8 cm long .............................................................................................................................................. 60
- 58. Corolla pink; inflorescence subumbelliform, congested, often with scattered hairs .............................................................................................................................................59
- Corolla white or very pale pink; inflorescence lax, cymose, glabrous ............................................................................................................................................. **I. lactifera**
- 59. Outer sepals < 7 long; inner sepals < 10 mm long; twining plant with stems 1 – 3 mm diam. and internodes 6 – 16 cm .......................................................................................................................................**I. aequatoriensis**
- Outer sepals > 7 mm long, inner sepals > 12 mm long; trailing or ascending herb with stems 2 – 6 mm diam. and internodes 2 – 10 cm ....................................................................................................................................... **I. batatas**
- 60. Sepals oblong-lanceolate, < 7 mm long, the margins whitish .......................................................................................................................................**I. tricolor**
- Sepals suborbicular, elliptic, > 7 mm long, ovate or oblong-elliptic, lacking white margins ................................................................................................................................................61
- 61. Sepals very unequal, the outer conspicuously shorter than the inner.......................................................................................................................................**I. squamosa**
- Sepals equal or nearly so ....................................................................................................................................... 62
- 62. Sepals > 10 mm long ....................................................................................................................................... 63
- Sepals < 10 mm long ....................................................................................................................................... 64
- 63. Sepals oblong-elliptic, clearly longer than broad, often translucent; inflorescence of simple axillary cymes ....................................................................................................................................... **I. chondrosepala**
- Sepals ovate to broadly oblong-elliptic, scarcely longer than broad, thick, often reddish; inflorescence usually of compound cymes, becoming somewhat paniculate ....................................................................................................................................... **I. philomega**
- 64. Sepals rounded, coriaceous, convex, nearly as broad as long ....................................................................................................................................... **I. batatoides**
- Sepals acute to obtuse, neither coriaceous nor convex, distinctly broader than long ....................................................................................................................................... 65
- 65. Inflorescence usually > 5-flowered, pedicels all erect; corolla c.7.5 cm long....................................................................................................................................... **I. quitensis**
- Inflorescence 1 – 5-flowered, the central pedicel reflexed; corolla 4.5 – 5 cm long. ....................................................................................................................................... **I. velardei** var. **aequatoriana**

Although there are relatively few species of *Ipomoea* endemic to Ecuador (the four newly described species in this paper, *I. habeliana, I. harlingii* and perhaps *I. aequatoriensis*), Ecuador shares a number of globally rare species with neighbouring Peru. These include *I. alexandrae*, *I. chrysocalyx*, *I. nationis* and *I. velardei.*
